# Supplementary material for: Complex Formation between VEGFR2 and the β2-Adrenoceptor
Source: Cell Chem Biol. 2019 Jun 20;26(6):830–841.e9. doi: 10.1016/j.chembiol.2019.02.014 (PMC6593180; doi:10.1016/j.chembiol.2019.02.014)
Supplement: Document S1. Figures S1–S6 and Table S1 [file mmc1.pdf]

Cell Chemical Biology, Volume *26*

## Supplemental Information

# Complex Formation between VEGFR2 and the $\beta_2$-Adrenoceptor

**Laura E. Kilpatrick, Diana C. Alcobia, Carl W. White, Chloe J. Peach, Jackie R. Glenn, Kris Zimmerman, Alexander Kondrashov, Kevin D.G. Pfleger, Rachel Friedman Ohana, Matthew B. Robers, Keith V. Wood, Erica K. Sloan, Jeanette Woolard, and Stephen J. Hill**

# Supplementary Information.

**Complex formation between VEGFR2 and the $\beta_2$-adrenoceptor.**

Laura E. Kilpatrick[1,2♯], Diana C. Alcobia[1,2,7♯], Carl W. White[1,2,5], Chloe J Peach[1,2], Jackie R Glenn[1,2], Kris Zimmerman[3]; Alexander Kondrashov[4], Kevin D. G. Pfleger[5,6], Rachel Friedman-Ohana[3], Matthew B. Robers[3], Keith V. Wood[3], Erica K Sloan[7,8,9], Jeanette Woolard[1,2,*], Stephen J. Hill[1,2,*]

**Supplementary Table 1 (Related to Figure 2).**

**Maximum BRET values ($BRET_{max}$ ; baseline corrected) for GPCR-GPCR and VEGFR2-GPCR heterodimers.**

| **Homodimers** | | | | |
|---|---|---|---|---|
| **Donor** | **Acceptor** | **$BRET_{MAX}$** | | **n** |
| NLuc-$A_3$R | SNAP-$A_3$R | 0.021 ± 0.001 | $p < 0.001$ | 5 |
| NLuc-$\beta_2$AR | SNAP-$\beta_2$AR | 0.088 ± 0.012 | $p < 0.001$ | 5 |
| **Heteromers** | | | | |
| **Donor** | **Acceptor** | **$BRET_{MAX}$** | | **n** |
| NLuc-VEGFR2 | SNAP-$A_3$R | - | NS | 5 |
| NLuc-VEGFR2 | SNAP-$\beta_2$AR | 0.015 ± 0.002 | $p< 0.01$ | 5 |

$BRET_{MAX}$ values for homodimers and NLuc-VEGFR2-heterodimers are taken from individual experiments summarized in Figure 2. Values are mean ± SEM from n separate experiments. Statistical analysis was performed using One-way ANOVA with Tukey's test and compares the $BRET_{MAX}$ value to the equivalent baseline BRET signal. NS=not significant.

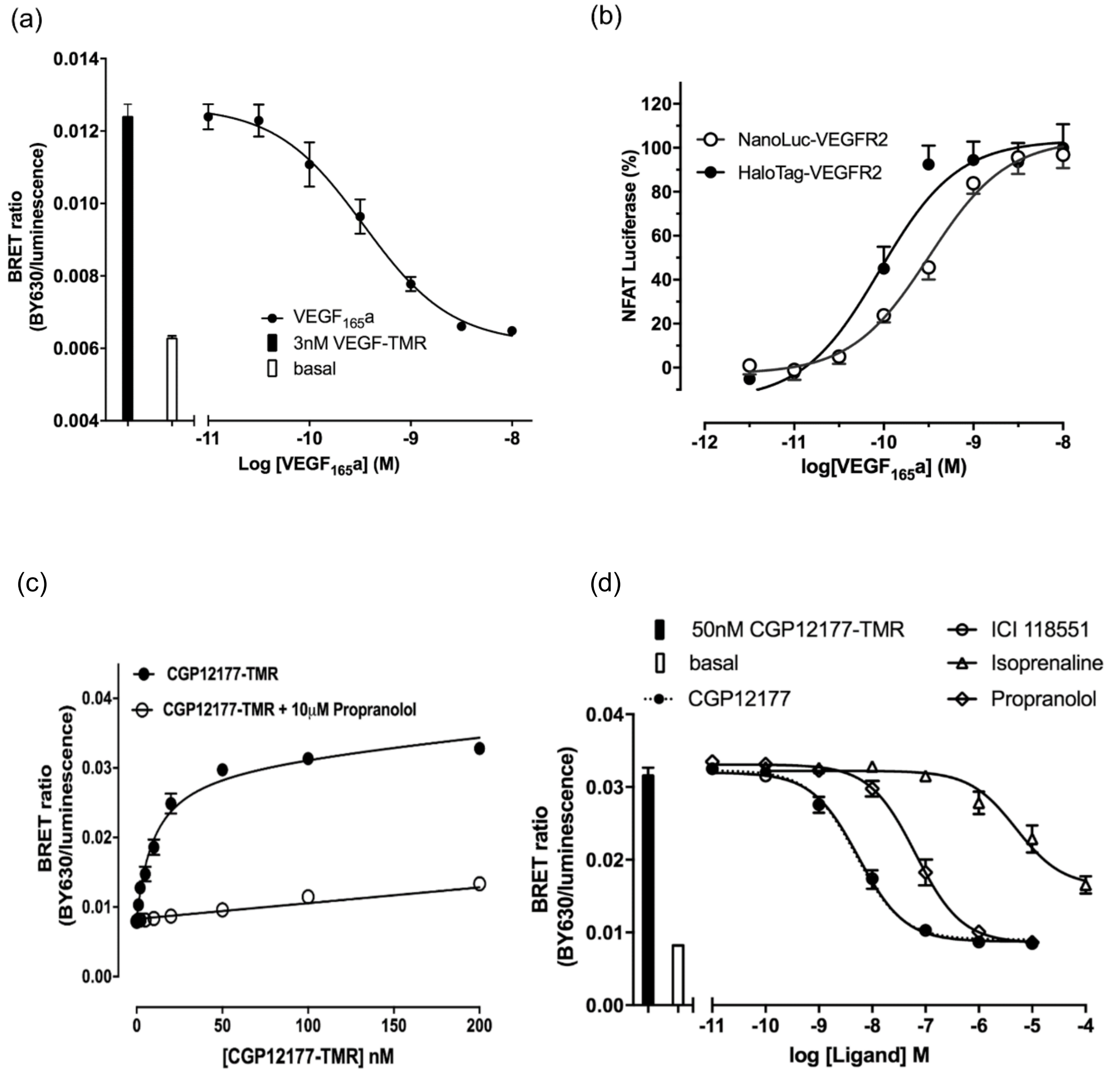


**Supplementary Figure S1. (Related to Figures 1 and 2).** (a,b) Characterization of the interaction between $VEGF_{165}a$ and VEGFR2 using ligand binding studies and functional responses in HEK293 cells. (a) Inhibition of the binding of 3nM $VEGF_{165}a$-TMR to NLuc-VEGFR2 by increasing concentrations of $VEGF_{165}a$. Data represent mean ± SEM from seven separate experiments, each performed in duplicate. (b) NFAT luciferase production in HEK293T cells stably expressing HaloTag-VEGFR2 or NLuc-VEGFR2 in response to stimulation (5h) by $VEGF_{165}a$. Data are mean ± SEM (5 independent experiments, duplicate wells) expressed as a percentage of the response to 10nM $VEGF_{165}a$. (c, d) Characterization of the ligand binding properties of BODIPY CGP12177-TMR. (c) NanoBRET saturation binding curves obtained for BODIPY CGP12177-TMR binding to NLuc-$\beta_2$-adenoceptors. Non-specific binding (open circles) was established by the addition of 10μM Propranolol. Values are mean ± SEM from five separate experiments each performed in triplicate. $pK_D$ of CGP12177-TMR was 7.98 ± 0.11 (n=5). (d) Inhibition of the binding of 50nM BODIPY CGP12177-TMR to NLuc-$\beta_2$-adrenoceptor by increasing concentrations of β-adrenoceptor

ligands. Data represent mean ± SEM from six separate experiments, each performed in duplicate. $pK_i$ values obtained for ICI 118551, propranolol, CGP12177 and isoprenaline were 9.06 ± 0.12, 7.97 ± 0.12, 8.88 ± 0.21 and 6.63 ± 0.24 respectively (n=6).

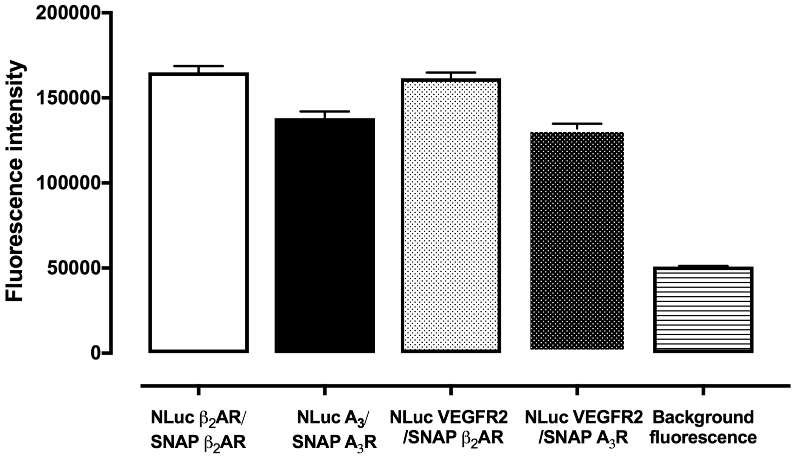


**Supplementary Figure S2 (related to Figure 2).** Comparison of expression levels of SNAP-tagged-β2-adrenoceptors and SNAP-tagged adenosine A3-receptors in transient transfection experiments involving NLuc-tagged GPCRs and NLuc-VEGFR2. Transient expression was performed with 50ng/well NLuc VEGFR2 or NLuc-GPCR and 100ng/well of SNAP-tag β2-adrenoceptor or adenosine A3-receptor cDNA. Background fluorescence was determined in cell transfects with 50ng/well NLuc-VEGFR2 and 100ng/well of pcDNA3.1-Zeo vector. Fluorescent intensity was determined on the PheraStar plate reader using excitation (485nm)/emission (520nm) filters from a 3x3 well scan matrix. Values represent mean ± SEM of 16 replicates in each of three independent experiments.

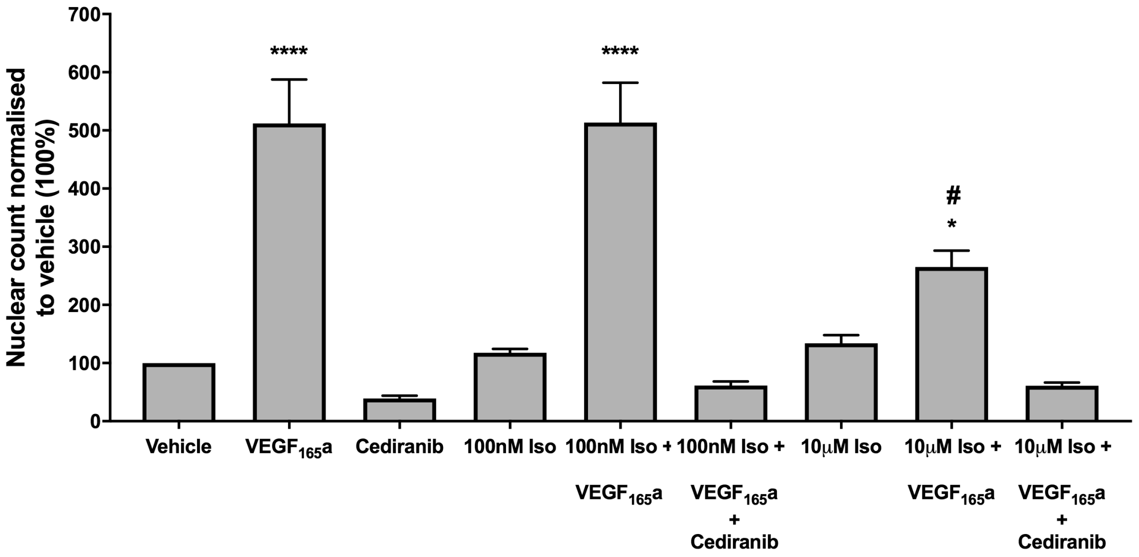


**Supplementary Figure S3. (Related to Figure 4c).** Effect of $VEGF_{165}$a and isoprenaline (Iso) stimulation on proliferation of human umbilical vein endothelial cells (HUVECs). HUVECs were serum starved in Medium 200/0.1% LVES serum for 24h prior to stimulation. On the day of the assay, HUVECs were stimulated in triplicate with $VEGF_{165}$a (3nM), isoprenaline (100nM or 10μM) or both ligands simultaneously in the presence or absence of the receptor tyrosine kinase inhibitor cediranib (1μM) for 48hr at 37°C/5% $CO_2$). Cediranib alone was used a negative control. HUVECs were then fixed using 3% paraformaldehyde in PBS and nuclei stained using H33342. Cells were imaged using a widefield IX Micro platereader and nuclei counted. Data are expressed as mean ± SEM pooled from 4 independent experiments and normalized to vehicle responses (100%). **** $p < 0.0001$ or * $p<0.05$ compared to vehicle and # $p<0.0001$ VEGF165a v Iso+VEGF165a (one-way ANOVA with Tukey's multiple comparison test).

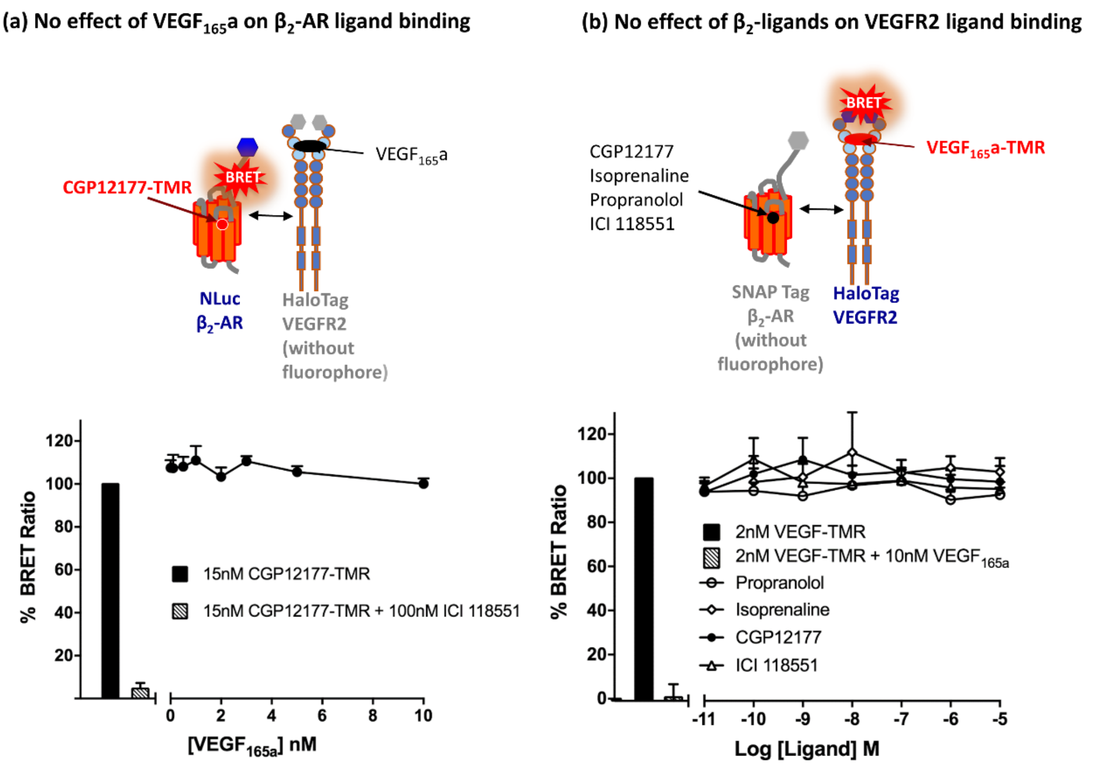


**Supplementary Figure S4**. **(Related to Figure 2).** No evidence for cooperativity across $\beta_2$-adrenoceptor-VEGFR2 interfaces provided by ligand-binding studies using a fluorescent ligand for the $\beta_2$-adrenoceptor (a; BODIPY CGP 12177-TMR) or the fluorescent $VEGF_{165}$a-TMR ligand for VEGFR2 (b; $VEGF_{165}$a-TMR). HEK293 cells were transiently transfected with a 1:2 cDNA ratio (0.05:0.1µg/well) of donor (NLuc-VEGFR2, NLuc-$\beta_2$-adrenoceptor) to acceptor (SNAP-Tag-$\beta_2$-adrenoceptor or HaloTag-VEGFR2) constructs. (a) NLuc-$\beta_2$-adrenoceptor and HaloTag-VEGFR2; (b) NLuc-VEGFR2 and SNAP-Tag-$\beta_2$-adrenoceptor. BRET was measured between donor NLuc and acceptor fluorescent ligands. Data are mean ± SEM from five separate experiments, each performed in duplicate.

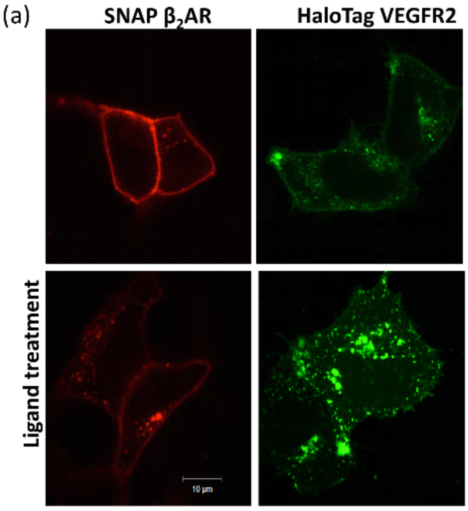


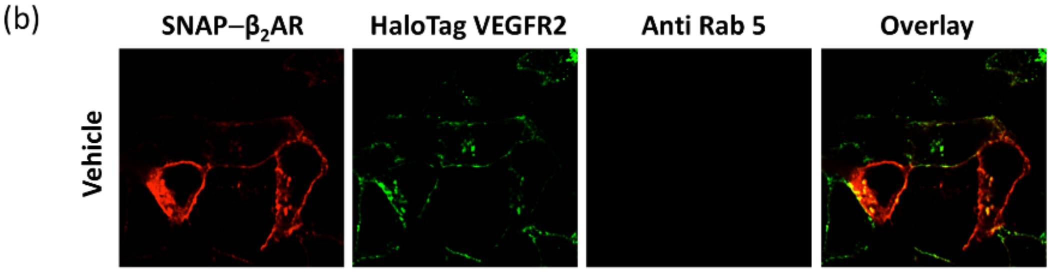


**Supplementary Figure S5. (Related to Figure 6).** (a) Live cell imaging showing the effect of agonist stimulation on the cellular location of $\beta_2$-adrenoceptors or VEGFR2 when expressed alone. Confocal imaging (Zeiss LSM 710) of live HEK293 cells transiently transfected with 0.25µg/well of SNAP-Tag-$\beta_2$AR or HaloTag VEGFR2 cDNAs, under unstimulated conditions (vehicle) or after treatment with 10µM isoprenaline (SNAP-$\beta_2$AR) or 10nM VEGF$_{165}$a ligands (HaloTag VEGFR2; 60min at 37°C). (b) Control experiments of secondary antibody only labelling of Rab 5 early endosomes. HEK293 cells transiently co-transfected with 0.5µg/well HaloTag VEGFR2 (green) and 0.5µg/well SNAP-Tag $\beta_2$AR (red) cDNAs, under unstimulated conditions (vehicle) (30min at 37°C). Cells were fixed using 3% PFA/PBS, permeabilised using Triton-X-100 (0.025% in PBS). Cells were then incubated with a secondary antibody (donkey anti rabbit AlexaFluor 546) in the absence of the primary antibody specific for Rab 5 endosomal compartments (rabbit anti Rab 5 monoclonal antibody). The overlay of HaloTag VEGFR2 and SNAP-Tag $\beta_2$AR expression with anti-Rab 5 labelling is shown. Data are representative of 3 individual experiments and were performed in parallel with those in Figure 6b.

(a)

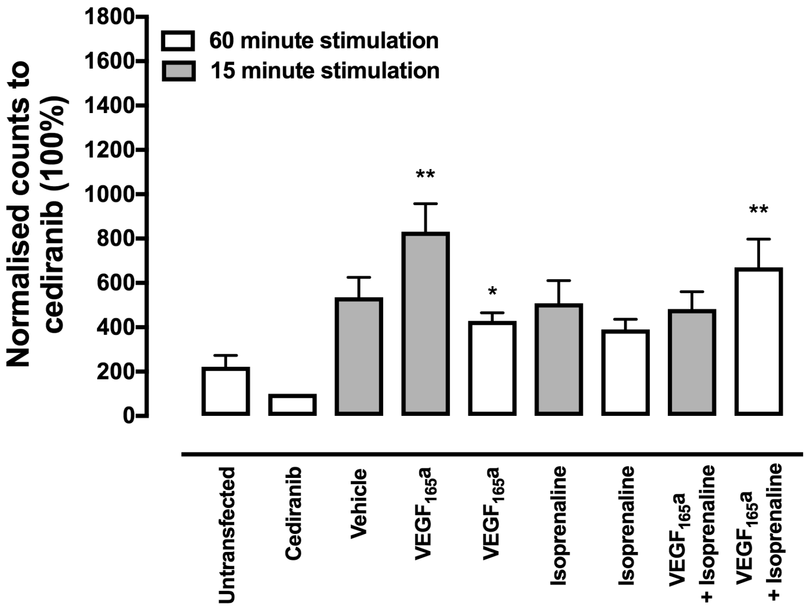


(b)

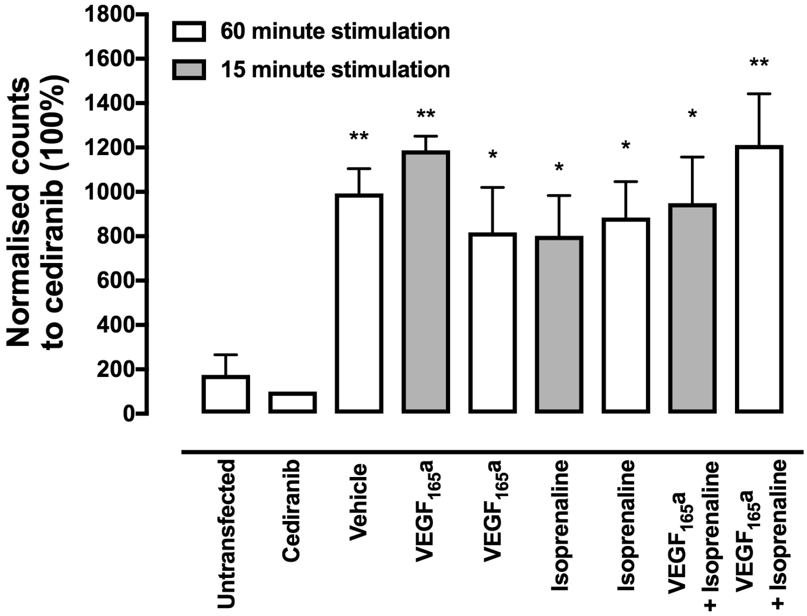


**Supplementary Figure S6 (related to Figure 7).** Probing the effect of isoprenaline stimulation on VEGFR2 activation status. HEK293 cells were transiently transfected with (a) HaloTag VEGFR2 or (b) HaloTag VEGFR2 and SNAP-Tag $\beta_2$-adrenoceptors (0.05μg/well of each construct) and stimulated with $VEGF_{165}a$ (3nM), isoprenaline (100nM) or both ligands simultaneously for 15 (grey bars) or 60min (white bars) at 37°C (5 replicates per stimulation). The receptor tyrosine kinase inhibitor cediranib was used as a negative control. Cells were fixed using 3% PFA/PBS and permeabilised using Triton-X-100 (0.025%). Immunolabelling

of the VEGFR2 tyrosine residue Y1212 was used to confirm the activation status of VEGFR2. Cell nuclei were stained using H33342 and images acquired using the IX Micro widefield platereader. Data were normalized as fold increase over cediranib responses (100%; mean ± SEM) and are pooled from 3 independent experiments. * ($p<0.05$) or ** ($p<0.01$) Compared to the cediranib control (One-way ANOVA with post hoc Tukey test).
