# Supplementary material for: Complex Formation between VEGFR2 and the β2-Adrenoceptor
Source: Cell Chem Biol. 2019 Jun 20;26(6):830–841.e9. doi: 10.1016/j.chembiol.2019.02.014 (PMC6593180; doi:10.1016/j.chembiol.2019.02.014)
Supplement: Document S2. Article plus Supplemental Information [file mmc2.pdf]

Article

Cell Chemical Biology

Complex Formation between VEGFR2 and the β_2-Adrenoceptor

Graphical Abstract

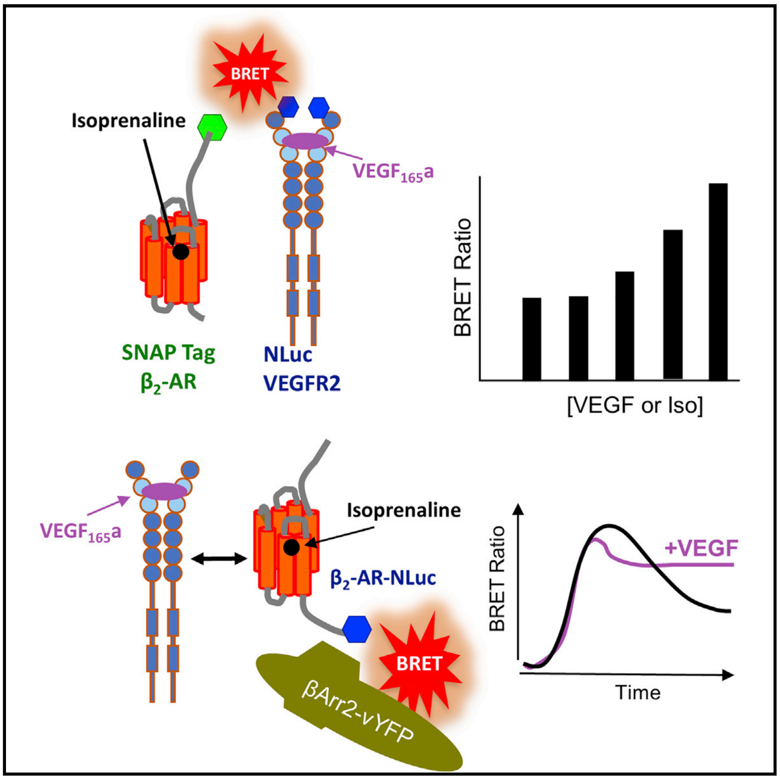

Highlights

- NanoBRET can monitor dimerisation of VEGFR2 and β_2-adrenoceptors in living cells
- Formation of VEGFR2/β_2-adrenoceptor complexes was enhanced by agonist stimulation
- β_2-Adrenoceptor/VEGFR2 complexes were internalized by agonist treatment to endosomes
- Coupling of β_2-adrenoceptors to β-arrestin2 was prolonged by VEGFR2 activation

Authors

Laura E. Kilpatrick, Diana C. Alcobia, Carl W. White, ..., Erica K. Sloan, Jeanette Woolard, Stephen J. Hill

Correspondence

jeanette.woolard@nottingham.ac.uk (J.W.),
stephen.hill@nottingham.ac.uk (S.J.H.)

In Brief

Kilpatrick et al. have used bioluminescence resonance energy transfer (BRET) and VEGFR2 tagged with NanoLuc luciferase, to demonstrate that oligomeric complexes involving VEGFR2 and β_2-adrenoceptors can be generated in both cell membranes and intracellular endosomes. These complexes are agonist sensitive and retain their ability to couple to intracellular signaling proteins.

Kilpatrick et al., 2019, Cell Chemical Biology *26*, 830–841
June 20, 2019 © 2019 The Author(s). Published by Elsevier Ltd.
https://doi.org/10.1016/j.chembiol.2019.02.014

CellPress

Cell Chemical Biology
Article

Complex Formation between VEGFR2 and the β_2-Adrenoceptor

Laura E. Kilpatrick,[1,2,10] Diana C. Alcobia,[1,2,7,10] Carl W. White,[1,2,5] Chloe J. Peach,[1,2] Jackie R. Glenn,[1,2] Kris Zimmerman,[3] Alexander Kondrashov,[4] Kevin D.G. Pfleger,[5,6] Rachel Friedman Ohana,[3] Matthew B. Robers,[3] Keith V. Wood,[3] Erica K. Sloan,[7,8,9] Jeanette Woolard,[1,2,11,*] and Stephen J. Hill[1,2,11,12,*]

[1]Division of Physiology, Pharmacology & Neuroscience, School of Life Sciences, University of Nottingham, Nottingham NG7 2UH, UK
[2]Centre of Membrane Proteins and Receptors, University of Birmingham and University of Nottingham, The Midlands, UK
[3]Promega Corporation, Madison, WI 53711, USA
[4]Wolfson Centre for Stem Cells, Tissue Engineering & Modelling (STEM), Centre for Biomolecular Sciences, University of Nottingham, Nottingham NG7 2RD, UK
[5]Harry Perkins Institute of Medical Research and Centre for Medical Research, The University of Western Australia, Nedlands, Perth, WA 6009, Australia
[6]Dimerix Limited, Nedlands, Perth, WA 6009, Australia
[7]Drug Discovery Biology, Monash Institute of Pharmaceutical Sciences, Monash University, Parkville, Melbourne, VIC 3052, Australia
[8]Cousins Center for Neuroimmunology, Semel Institute for Neuroscience and Human Behavior, Jonsson Comprehensive Cancer Center, UCLA AIDS Institute, University of California, Los Angeles, CA 90095, USA
[9]Division of Surgical Oncology, Peter MacCallum Cancer Centre, Victorian Comprehensive Cancer Centre, 305 Grattan Street, Melbourne, VIC 3000, Australia
[10]These authors contributed equally
[11]Senior author
[12]Lead Contact
*Correspondence: jeanette.woolard@nottingham.ac.uk (J.W.), stephen.hill@nottingham.ac.uk (S.J.H.)
https://doi.org/10.1016/j.chembiol.2019.02.014

SUMMARY

Vascular endothelial growth factor (VEGF) is an important mediator of endothelial cell proliferation and angiogenesis via its receptor VEGFR2. A common tumor associated with elevated VEGFR2 signaling is infantile hemangioma that is caused by a rapid proliferation of vascular endothelial cells. The current first-line treatment for infantile hemangioma is the β-adrenoceptor antagonist, propranolol, although its mechanism of action is not understood. Here we have used bioluminescence resonance energy transfer and VEGFR2 genetically tagged with NanoLuc luciferase to demonstrate that oligomeric complexes involving VEGFR2 and the β_2-adrenoceptor can be generated in both cell membranes and intracellular endosomes. These complexes are induced by agonist treatment and retain their ability to couple to intracellular signaling proteins. Furthermore, coupling of β_2-adrenoceptor to β-arrestin2 is prolonged by VEGFR2 activation. These data suggest that protein-protein interactions between VEGFR2, the β_2-adrenoceptor, and β-arrestin2 may provide insight into their roles in health and disease.

INTRODUCTION

Vascular endothelial growth factor A (VEGF-A) is an important mediator of endothelial cell proliferation and angiogenesis (Ferrara, 2009; Shibuya, 2011; Musumeci et al., 2012). VEGF-A mediates its effects on endothelial cells predominantly via the receptor tyrosine kinase (RTK) VEGF receptor 2 (VEGFR2), which also represents an important drug target for cancer angiogenesis (Ferrara, 2009; Claesson-Welsh and Welsh, 2013; Peach et al., 2018a). VEGFR2 signaling is elevated in infantile hemangioma due to the rapid proliferation of vascular endothelial cells during early infancy (Ou et al., 2014). The current first-line treatment for infantile hemangioma is the β-adrenoceptor antagonist, propranolol, although its mechanism of action is not fully understood (Léauté-Labrèze et al., 2008; Ozeki et al., 2016; Stiles et al., 2012; Mulcrone et al., 2017). The therapeutic effect of propranolol is mediated by antagonism of the β_2-adrenoceptor (a G protein-coupled receptor [GPCR]) and appears to result from reduced VEGF-A expression and cell proliferation (Ozeki et al., 2016; Stiles et al., 2012; Mulcrone et al., 2017; Park et al., 2011). β_2-Adrenoceptor activation has also been reported to play a critical role in mediating stress-induced metastasis in breast cancer (Mulcrone et al., 2017; Sloan et al., 2010; Chang et al., 2016) and cancer angiogenesis in prostate cancer (Hulsurkar et al., 2017). For example, skeletal colonization by breast cancer cells is stimulated by a β_2-adrenoceptor- and VEGF-dependent neo-angiogenic switch (Mulcrone et al., 2017).

In addition to GPCR agonists eliciting changes in the expression of growth factors (such as VEGF-A), there is accumulating evidence for complex interactions between their cognate receptors (Luttrell et al., 1999; George et al., 2013; Pyne and Pyne, 2011; Liebmann, 2011). Three different mechanisms of GPCR-RTK interaction have been identified: (1) GPCR activation of matrix metalloproteases leading to the shedding of heparin-binding growth factors (e.g., heparin-binding epidermal growth factor) and the subsequent activation of their receptors (Daub et al.,

This is an open access article under the CC BY license (http://creativecommons.org/licenses/by/4.0/).

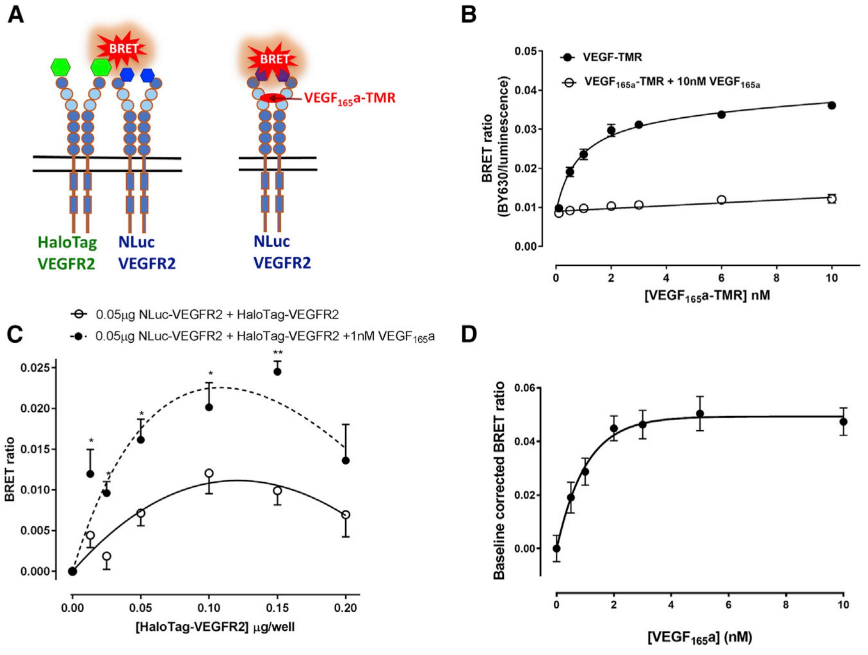

Figure 1. Using NanoBRET to Characterise the Formation of VEGFR2 Homodimers and Ligand Binding at VEGFR2

(A) Schematic representation of the use of NanoBRET to investigate the interaction between NLuc-tagged VEGFR2 (NLuc-VEGFR2) and HaloTag-VEGFR2, or the binding of a fluorescent analog of VEGF$_{165}$a to NLuc-VEGFR2.

(B) NanoBRET saturation binding curves obtained for VEGF$_{165}$a-TMR binding to NLuc-tagged VEGFR2. HEK293 cells stably transfected with NLuc-VEGFR2 were treated for 60 min with increasing concentrations of VEGF$_{165}$a-TMR (filled circles). Non-specific binding (open circles) was determined in the presence of 10 nM VEGF$_{165}$a. Values are means ± SEM from four separate experiments each performed in triplicate. pK_D of VEGF$_{165}$a-TMR was 9.00 ± 0.16 (n = 4).

(C) BRET experiments investigated the constitutive and ligand-induced dimerization of VEGFR2. HEK293 cells were transiently transfected with a fixed concentration of donor NLuc-VEGFR2 cDNA (0.05 µg/well) and increasing concentrations of acceptor HaloTag-VEGFR2 cDNA. Cells were treated with either vehicle (open circles) or 1 nM VEGF$_{165}$a (filled circles) for 60 min at 37°C. Duplicate measurements were made for each condition in each individual experiment and values shown are the means ± SEM obtained in seven separate experiments. *$p < 0.05$; **$p < 0.001$; student's t test.

(D) HEK293T cells were transiently transfected with 0.05 µg/well HaloTag VEGFR2 cDNA and 0.025 µg/well of NLuc-VEGFR2 cDNA and treated with increasing concentrations of VEGF$_{165}$a for 60 min at 37°C. Values are means ± SEM obtained in six separate experiments.

1997; Prenzel et al., 1999; Hart et al., 2005); (2) GPCR mediated transactivation and phosphorylation of RTKs following activation of intracellular signaling cascades (Luttrell et al., 1999; Thuringer et al., 2002; Zajac et al., 2011); and (3) GPCR-RTK oligomerization (Liebmann, 2011; Bergelin et al., 2010). With respect to receptor oligomerization, it is well established that RTKs exist as preformed dimers or can be induced to dimerize following the binding of their cognate ligand (Bessman et al., 2014a; 2014b; Freed et al., 2015). Studies with purified extracellular domains of VEGFR2 have provided evidence for VEGF-A-induced homodimerization (Dosch and Ballmer-Hofer, 2010; Brozzo et al., 2012; Markovic-Mueller et al., 2017). However, recent work has also suggested that VEGFR2 can form dimers in the absence of VEGF (Sarabipour et al., 2016). Furthermore, signaling complexes have been reported between VEGFR2 and the GPCR sphingosine-1-phosphate receptor in thyroid carcinoma cells (Bergelin et al., 2010). This raises the prospect that VEGFR2 may form oligomeric complexes with β_2-adrenoceptors and provide a potential mechanism of action for the therapeutic benefit of propranolol in hemangioma.

Here we have used bioluminescence resonance energy transfer (BRET) with VEGFR2 genetically tagged with the NanoLuc (NLuc) luciferase (Stoddart et al., 2015; 2018; Kilpatrick et al., 2017) to investigate complex formation between VEGFR2 and the β_2-adrenoceptor in living cells.

RESULTS

Constitutive and VEGF$_{165}$a-Induced VEGFR2 Homodimerization

To monitor ligand binding and receptor dimerization of VEGFR2 using BRET, we tagged VEGFR2 on its N terminus with the bright, small (19.1 kDa) NLuc luciferase (Stoddart et al., 2015; Kilpatrick et al., 2017; Peach et al., 2018b) (see scheme in Figure 1A). Use of a fluorescent analog of VEGF$_{165}$a enabled ligand binding to be monitored by NanoBRET in HEK293 cells stably expressing NLuc-VEGFR2 (Figure 1B), and to demonstrate that the NLuc-tagged VEGFR2 retains its high affinity for VEGF$_{165}$a (Figure S1A). We were also able to show that constitutive VEGFR2 dimers are formed in HEK293 cells by co-transfecting cells with NLuc-VEGFR2 and an N-terminal HaloTag-labeled VEGFR2 (Figure 1C). In these experiments HEK293 cells were transiently transfected with a fixed concentration of donor NLuc-VEGFR2 cDNA and increasing concentrations of acceptor HaloTag VEGFR2 cDNA. The BRET signal clearly saturated (as would be expected for a specific protein-protein interaction [Mercier et al., 2002]) and then began to decrease at the highest HaloTag VEGFR2 cDNA concentration used, probably due to the concentration of protein not being linearly related to cDNA concentration at high amounts. Importantly, these effects were significantly enhanced when cells were treated with 1 nM VEGF$_{165}$a for 60 min at 37°C (Figure 1C). Analysis of the effect of increasing concentrations of VEGF$_{165}$a on the homodimerization obtained when 0.05 µg/well HaloTag VEGFR2 cDNA was transiently transfected with 0.025 µg/well of NLuc-VEGFR2 cDNA showed a clear saturable effect with a half maximal effective concentration (pEC_{50}; $-\log EC_{50}$) for VEGF$_{165}$a of 8.81 ± 0.20 (n = 6; Figure 1D). A similar effect was observed when 0.05µg/well HaloTag VEGFR2 cDNA was transiently transfected into a stable NLuc-VEGFR2 cell line (Kilpatrick et al., 2017) yielding a pEC_{50} for VEGF$_{165}$a of 9.40 ± 0.28 (n = 4). These values are very similar to the binding affinity of VEGF$_{165}$a determined from NanoBRET binding (pK_i = 10.17 ± 0.09, n = 7; Figure S1A) and to the pEC_{50} values for VEGF$_{165}$a

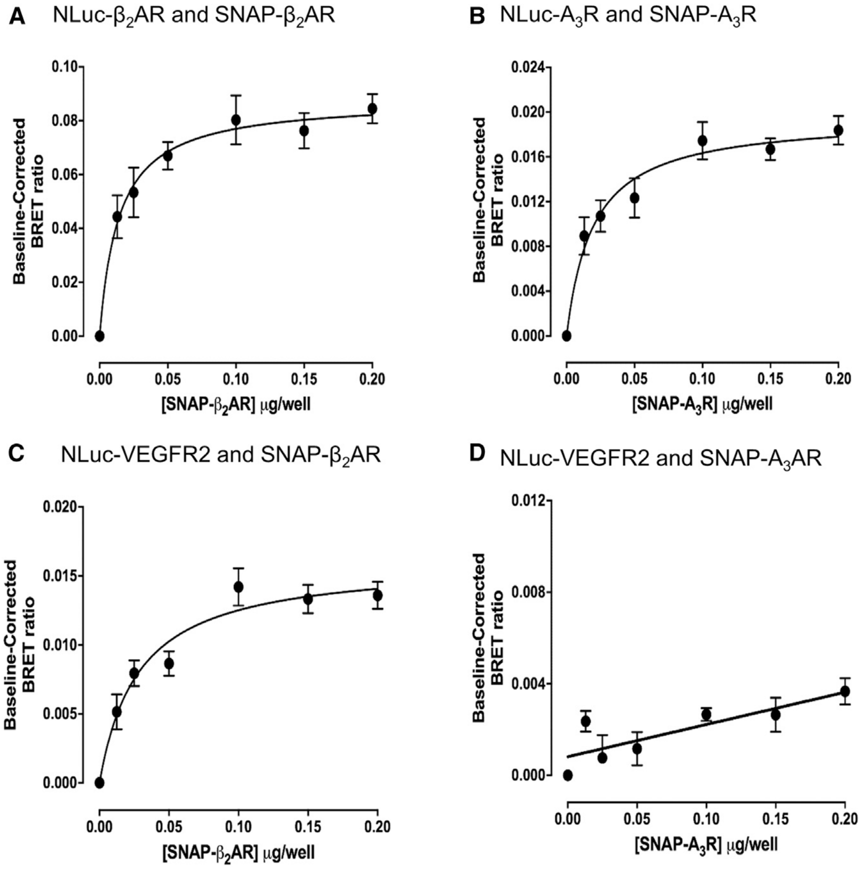

Figure 2. BRET Experiments Investigating GPCR Homo-Dimerization and Complex Formation between GPCRs and VEGFR2

(A and B) GPCR homodimer formation was investigated using transient transfection with NLuc-GPCR cDNA (0.05 μg/well) and increasing concentrations of SNAP-tagged GPCR cDNA for (A) the β_2-adrenoceptor (β_2AR) or (B) the adenosine A_3-receptor (A_3R). Data are means ± SEM from five separate experiments, each performed in duplicate.

(C and D) Complex formation between VEGFR2 and GPCRs. HEK293 cells were transfected with NLuc-VEGFR2 cDNA (0.05 μg/well) and increasing concentrations of SNAP-tagged GPCR cDNA for (C) the β_2-adrenoceptor or (D) the adenosine A_3-receptor. Data are means ± SEM from five separate experiments, each performed in duplicate.

obtained using an NFAT reporter assay in cells expressing either NLuc-VEGFR2 or HaloTag-VEGFR2 (9.50 ± 0.06 and 10.00 ± 0.14 for NLuc-VEGFR2 and HaloTag-VEGFR2, respectively; n = 5 in each case; Figure S1B). These values were also similar to those previously reported for the wild-type VEGFR2 (pEC_{50} = 9.66 ± 0.05 [Carter et al., 2015]) and confirm that the N-terminal NLuc and HaloTag labels do not interfere with intracellular signaling or binding of $VEGF_{165}$a.

VEGFR2-GPCR Oligomeric Complexes

It is well established that many GPCRs can form homodimers (Ferré et al., 2014; Vischer et al., 2015; Parmar et al., 2016). Here, we have used transient expression of NLuc- and SNAP-tagged GPCR pairs to demonstrate that the two GPCRs (β_2-adrenoceptor and adenosine A_3 receptors) studied here can form homodimers that are detectable using BRET (Figures 2A and 2B). Transient transfection of donor NLuc-tagged GPCR cDNA and increasing concentrations of acceptor SNAP-tagged GPCR cDNA revealed a clear and statistically significant saturation of the BRET signal that was consistent with a specific protein-protein interaction (Figures 2A and 2B; Table S1). When NLuc-VEGFR2 cDNA was co-transfected with SNAP-tagged GPCR cDNA, evidence for a selective interaction with β_2-adrenoceptors was revealed (Figure 2C; Table S1). Clear saturation of the BRET signal was observed that was in keeping with close proximity (<10 nm [Mercier et al., 2002]) between VEGFR2 and the β_2-adrenoceptor (Figure 2C). Given the propensity for both VEGFR2 and the β_2-adrenoceptor to form homodimers, this may represent the formation of a larger oligomeric complex. In marked contrast, no evidence for a specific interaction between VEGFR2 and the adenosine A_3 receptor was observed (Figure 2D). The BRET signal increased linearly with increasing concentration of acceptor SNAP-tagged adenosine A_3-receptor cDNA which is consistent with a non-specific interaction caused by bystander BRET (Figure 2D) (Mercier et al., 2002). A comparison of the expression level of SNAP-tagged β_2-adrenoceptors and adenosine A_3 receptors in these transient transfection experiments confirmed that very similar levels of expression of the two GPCRs were achieved in the presence of NLuc-VEGFR2 (Figure S2).

A study of the impact of agonist stimulation on the formation of VEGFR2-β_2-adrenoceptor complexes (Figure 3) indicated that there was a significant concentration-dependent enhancement of VEGFR2-β_2-adrenoceptor complex formation induced by either $VEGF_{165}$a or isoprenaline (Figures 3C and 3D). Interestingly, in cells transfected with both VEGFR2 and β_2-adrenoceptor cDNA, $VEGF_{165}$a was still able to stimulate VEGFR2 dimerization and both basal and VEGF-stimulated VEGFR2 homodimerization was unaffected by co-stimulation with isoprenaline (Figures 3E and 3G). Similarly, $VEGF_{165}$a (Figure 3F) and isoprenaline treatment (Figure 3H) did not alter β_2-adrenoceptor homodimerization.

To ensure that VEGFR2-β_2-adrenoceptor oligomeric complexes were not a result of receptor overexpression, we took advantage of the endogenously expressed β_2-adrenoceptors in HEK293 cells. Using CRISPR/Cas9 genome-engineering, we generated HEK293 cells that expressed NLuc-β_2-adrenoceptors under the control of the native promoter. These studies showed a significant increase in the BRET ratio for NLuc-β_2-adrenoceptors expressed under the endogenous promoter and exogenously transfected with 0.01 μg/well HaloTag-VEGFR2 (fluorophore-labeled HaloTag-VEGFR2 compared with cells where the HaloTag-VEGFR2 was not labeled with fluorophore) (Figures 4A and 4B). This supports the observation of a specific VEGFR2-β_2-adrenoceptor oligomeric complex and

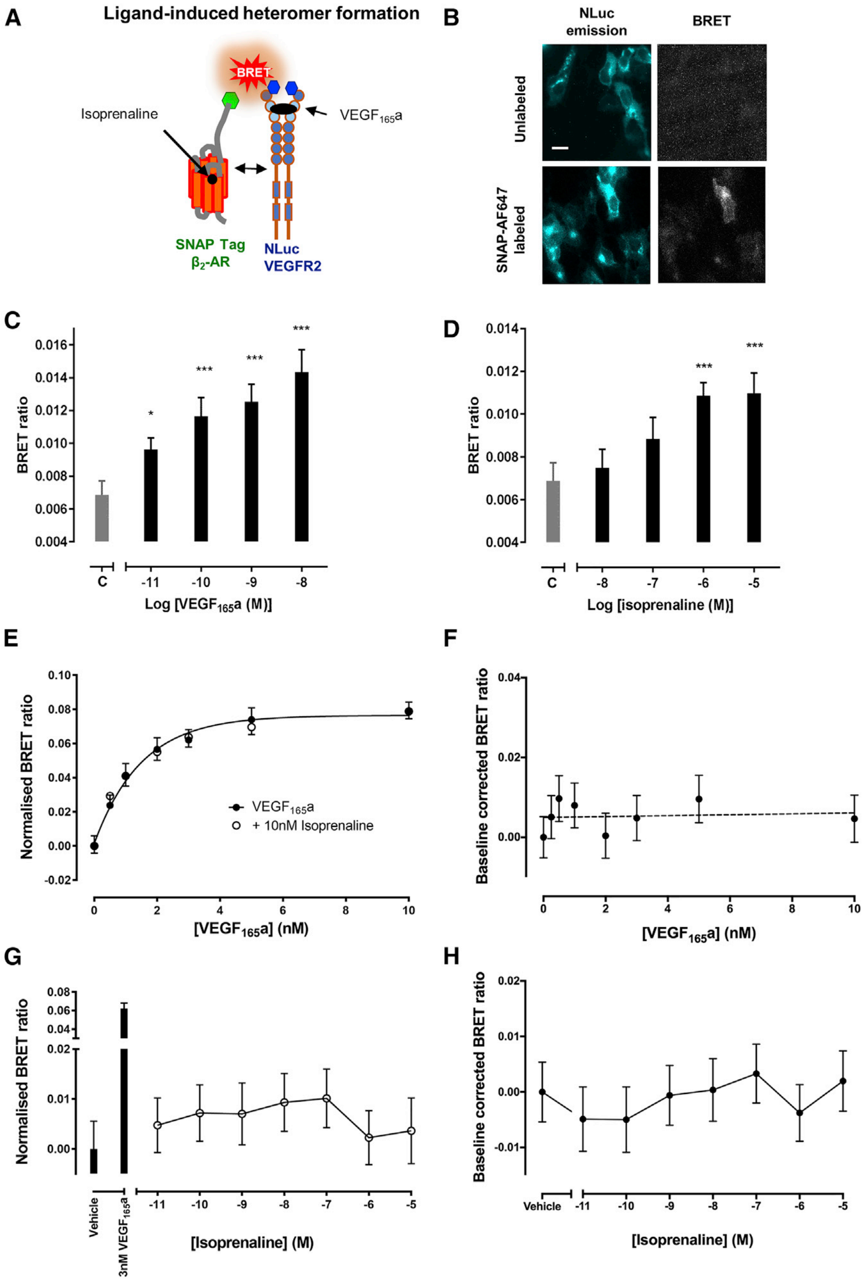

Figure 3. Effect of Agonist Stimulation on Receptor Oligomerization

(A) Schematic of experimental setup to investigate the effect of isoprenaline or VEGF$_{165}$a on receptor oligomerization measured using NanoBRET.

(B) Visualization of VEGFR2/β_2-adrenoceptor oligomers by NanoBRET using a luminescence LV200 Olympus microscope. HEK293 cells were transiently co-transfected to express NLuc-tagged-VEGFR2 and SNAP-tagged β_2-adrenoceptors. Sequential images were captured from unlabeled (top panels) or SNAP-surface AF647-labeled co-transfected cells (bottom panels). Sequential images were acquired by capturing DAPI channel, displayed in the left panels (donor detection; using a 438/24 nm emission filter, 5 s exposure time), followed by CY5 channel, displayed in the right panels (BRET-excited acceptor, using a 647 long-pass filter, 30 s exposure time). Scale bar represents 20 μm.

(C and D) HEK293 cells were transiently transfected with 0.05 μg/well NLuc-VEGFR2 and 0.10 μg/well SNAP-β_2-AR and treated for 1 h at 37°C with increasing concentrations of (C) VEGF$_{165}$a or (D) isoprenaline. Bar C corresponds to untreated (control) condition. Data are means ± SEM from five separate experiments, each performed in quadruplicate. **p < 0.005 or ***p < 0.001 compared with control (C) (two-way ANOVA with Dunnett's multiple comparison test).

(E and G) HEK293 cells were transiently transfected with 0.05 μg/well NLuc-VEGFR2, 0.05 μg/well HaloTag VEGFR2 and 0.05 μg/well SNAP-β_2-AR (unlabeled) and treated for 1 h at 37°C with increasing concentrations of VEGF$_{165}$a in the presence or absence of a fixed concentration of isoprenaline (10 nM) (E) or increasing concentrations of isoprenaline alone (G). Data are means ± SEM from five separate experiments each performed in quadruplicate.

(F and H) HEK293 cells were transiently transfected with 0.05 μg/well NLuc-β_2-AR 0.05 μg/well SNAP-β_2-AR and 0.05 μg/well HaloTag VEGFR2 (unlabeled) and treated for 1 h at 37°C with increasing concentrations of (F) VEGF$_{165}$a or (H) isoprenaline. Data are means ± SEM from five or six separate experiments each performed in quadruplicate.

also demonstrates that formation and detection of the complexes is independent of tag orientation.

Electroporation of human umbilical vein endothelial cells (HUVECs) = with NLuc-VEGFR2 also showed a significant increase in BRET ratio ($p < 0.05$) when the cells were co-transfected with SNAP-Tag-β_2-adrenoceptor cDNA (Figure 4C) suggesting that heterodimers can be formed in endothelial cells. To investigate further the potential for endogenous VEGFR2 and β_2-adrenoceptors to interact in HUVECs, we also investigated their ability (alone and in combination) to stimulate cell proliferation. As reported previously (Kilpatrick et al., 2017), 3 nM VEGF$_{165}$a was able to produce a large and significant enhancement of cellular proliferation that could be inhibited by the tyrosine kinase inhibitor, cediranib (Figure S3). A very small but not significant increase in cell number was observed with both high (10 μM) and low (100 nM) concentrations of isoprenaline alone (Figure S3). However, in the presence of 10 μM isoprenaline the response to 3 nM VEGF$_{165}$a was significantly attenuated (Figure S3).

We have previously reported that dimer formation can lead to negative cooperativity between the ligand binding sites of the two partners within an oligomeric complex (Gherbi et al., 2015; May et al., 2011). Therefore, to investigate the potential for negative cooperativity across oligomeric interfaces, we took advantage of our ability to measure ligand binding to NLuc-tagged receptors using BRET and fluorescent ligands (Stoddart et al., 2015; Kilpatrick et al., 2017). Clear saturable and high-affinity specific binding of the fluorescent ligand BODIPY CGP12177-TMR

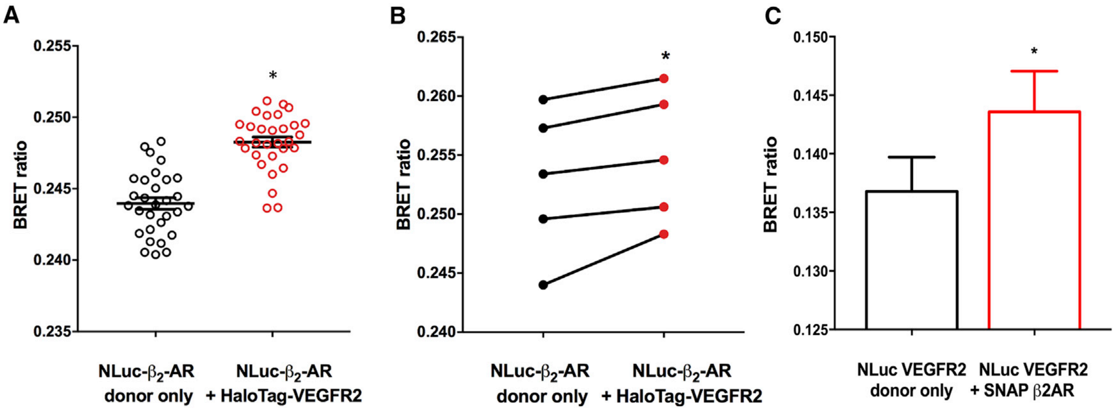

Figure 4. HEK293 Cells Expressing Gene-Edited NLuc-β_2-Adrenoceptor Labeled with HaloTag AF488 in the Absence or Presence of Transiently Transfected HaloTag-VEGFR2 (0.01 μg/well)

(A) Data from a single experiment performed with 36 replicates. *$p < 0.05$ (unpaired t test) compared with donor alone. Similar data were obtained in four separate experiments. In each repeat experiment significant differences ($p < 0.05$) were observed from donor alone apart from in one where $p = 0.07$.

(B) Mean paired data from the five separate experiments, each performed with 30 or 36 replicates. *$p < 0.05$ compared with donor only (paired two-tailed t test).

(C) Human umbilical vein endothelial cells (HUVECs) transiently transfected via electroporation with NLuc-VEGFR2 in the presence or absence of SNAP-β_2-AR. Data were pooled from eight independent transfections (four to eight replicates per experiment for each transfection) and expressed as mean ± SEM. *$p < 0.05$ compared with NLuc-VEGFR2 donor only (paired two-tailed t test; $p = 0.035$).

to β_2-adrenoceptors (K_D = 67.2 ± 3.3 nM, n = 4; Figure S1C) was observed, which was competitively antagonized by non-fluorescent β_2-adrenoceptor ligands (Figure S1D). However, no significant alteration in the binding of BODIPY CGP12177-TMR or VEGF$_{165}$a-TMR to their cognate receptors was observed with the addition of ligands for the corresponding receptor heteromer partner (Figure S4). Thus, VEGF$_{165}$a did not influence the binding of BODIPY CGP12177-TMR to the β_2-adrenoceptor (Figure S4A) and ICI118551, CGP12177, propranolol, and isoprenaline did not influence binding of VEGF$_{165}$a-TMR to VEGFR2 (Figure S4B).

Functional Impact of VEGFR2-β_2-Adrenoceptor Complexes on β_2-Adrenoceptor Activation

To investigate the potential impact of VEGFR2 complexes on β_2-adrenoceptor signaling and function, we first studied whether the VEGFR2-β_2-adrenoceptor oligomeric complex altered the extent to which an active β_2-adrenoceptor could engage with a conformation-sensitive single-domain nanobody (Nb80) that has previously found use in structural studies as a Gs-alpha surrogate protein (Rasmussen et al., 2011; Steyaert and Kobilka, 2011). Here we used a GFP-tagged version of Nb80 (Irannejad et al., 2013) in conjunction with a β_2-adrenoceptor tagged on its C terminus with NLuc, to establish a NanoBRET assay in living cells to monitor engagement (based on proximity) between an activated β_2-adrenoceptor and cytosolic Nb80-GFP. Isoprenaline (10 μM) stimulation of stable Nb80-GFP-expressing HEK293 cells, transiently transfected with β_2-adrenoceptor-NLuc, produced a rapid and significant binding of Nb80-GFP to β_2-adrenoceptors (Figure 5A; $p < 0.001$) confirming the ability of Nb80-GFP to detect active conformations of the β_2-adrenoceptor. To investigate the impact of VEGFR2 on β_2-adrenoceptor signaling, we also transfected cells with either HaloTag-VEGFR2 or an empty control vector. In cells additionally transfected with the control empty vector, isoprenaline stimulated the formation of a complex between the β_2-adrenoceptor and Nb80 (log EC_{50} = −7.91 ± 0.11, n = 6; Figure 5B). This response was competitively antagonized by the high-affinity β_2-selective antagonist, ICI 118551 (log K_D = −9.30 ± 0.20, n = 6; Figure 5B). In the presence of VEGFR2, the response to isoprenaline was unaltered, compared with control vector (Figure 5B). The successful expression of HaloTag-VEGFR2 in these cells was confirmed at the end of the experiment by labeling cells with the HaloTag substrate (data not shown).

Cellular Location of VEGFR2-β_2-Adrenoceptor Complexes

Confocal imaging of SNAP-tagged β_2-adrenoceptors and HaloTag-tagged VEGFR2 labeled with cell-impermeable dyes showed that, under basal conditions, cell surface β_2-adrenoceptors largely remained on the plasma membrane when expressed alone (Figure S5A). However, as reported previously (Kilpatrick et al., 2017), HEK293 cells expressing HaloTag-VEGFR2 showed evidence of constitutive receptor internalization (Figure S5A). Following treatment with 10 nM VEGF$_{165}$a, VEGFR2 internalization was markedly increased (Figure S5A). β_2-adrenoceptor internalization was also stimulated by isoprenaline (10 μM) (Figure S5A). Following co-transfection of HEK293 cells with HaloTag-VEGFR2 and SNAP-Tag β_2-adrenoceptor cDNA, the constitutive internalization of VEGFR2 appeared to be accompanied by a low level constitutive internalization of the β_2-adrenoceptor (Figure 6A). Isoprenaline (10 μM) stimulated a large internalization of the β_2-adrenoceptor that was co-localized with internalized VEGFR2 (Figure 6A). Stimulation of co-transfected cells with 10 nM VEGF$_{165}$a produced an enhanced internalization of VEGFR2 that was also accompanied by a partial internalization of cell surface β_2-adrenoceptors (Figure 6A). Co-localized receptors were readily detected in intracellular Rab5-positive endosomes under both basal or agonist-stimulated (VEGF$_{165}$a or isoprenaline) conditions (Figure 6B). Control experiments for Rab5 localization are shown in Figure S5B. Structured illumination

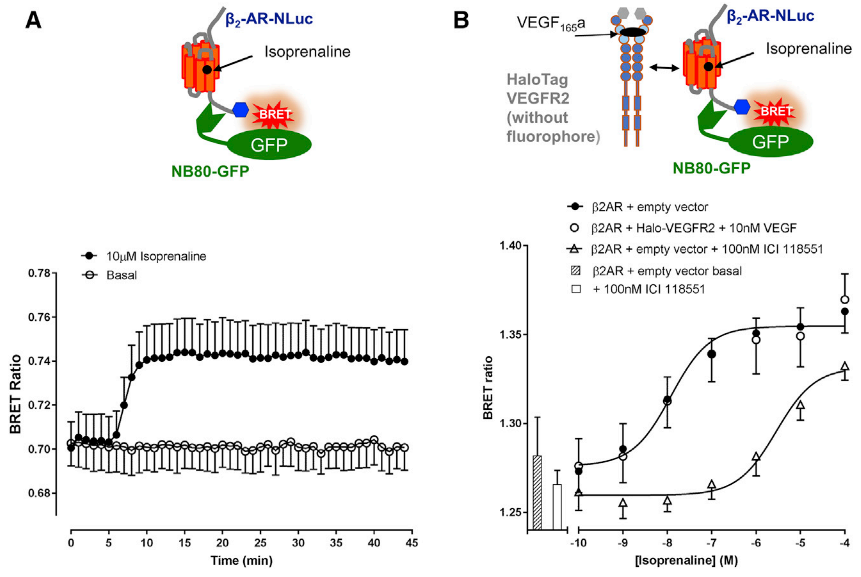

Figure 5. Investigation of the Activation Status of β_2-AR Using Nb80-GFP

(A) HEK293 cells stably expressing Nb80-GFP were transfected with 0.025 μg/well β_2-AR-NLuc cDNA and stimulated with isoprenaline (10 μM) or vehicle control added at 5 min. A significant ($p < 0.001$) increase in BRET ratio (relative to time zero) was observed with isoprenaline from 8 min onward (two-way ANOVA with repeated measures and Bonferroni's multiple comparison test). Data are means ± SEM from five separate experiments each performed in triplicate.

(B) HEK293 cells stably expressing Nb80-GFP were transiently co-transfected with 0.025 μg/well β_2-AR-NLuc cDNA and either 0.025 μg/well empty vector (pcDNA3.1) or 0.025 μg/well HaloTag-VEGFR2 cDNA. Cells co-transfected with empty vector were treated with increasing concentrations of isoprenaline, in the presence or absence of 100 nM ICI 118551, and cells co-transfected with HaloTag-VEGFR2 were co-stimulated with 10 μM isoprenaline and 10 nM $VEGF_{165}a$. Bars correspond to untreated and 100 nM ICI 118551-treated conditions. Data are means ± SEM from six separate experiments each performed in triplicate.

super-resolution microscopy (SIM) confirmed that there was co-localization of HaloTag VEGFR2 and SNAP-Tag β_2-adrenoceptors at intracellular sites, which was elevated after agonist stimulation (Figures 6C and 6D). The Fiji (ImageJ) analysis program CoLoc2 was applied to these SIM images to determine the extent of HaloTag VEGFR2 (green; HaloTag AF488 substrate) and SNAP-Tag β_2-adrenoceptor (red; SNAP-AF647 substrate) fluorescence co-localization (Figure 6D). Circular regions of interest (ROI) were placed on areas of fluorescence at the plasma membrane or intracellular regions (12–15 ROI; Figure 6E). Pearson's correlation coefficient values were obtained from each ROI, pooled, and expressed as mean ± SEM. A Pearson's correlation coefficient of +1 is indicative of a perfect co-occurrence between the fluorophores of interest (AF488 and AF647, respectively).

Interaction with β-Arrestin

Receptor internalization and signaling from endosomes are regulated by β-arrestin scaffolding proteins (β-arrestin 1 and 2; also known as arrestin2 and 3 [Shenoy and Lefkowitz, 2011; Laporte et al., 2000; Luttrell et al., 2001; Eichel and von Zastrow, 2018]). Here we have used the Receptor Heteromer Investigation Technology approach (Jaeger et al., 2014), with β-arrestin2-Venus-YFP in combination with a β_2-adrenoceptor tagged on its C terminus with NLuc, to investigate using NanoBRET the effect of VEGFR2 activation on isoprenaline-induced receptor engagement with β-arrestin2 in cells co-expressing unlabeled HaloTag-VEGFR2 (Figure 7A). Stimulation with 10 μM isoprenaline alone induced β-arrestin2-Venus-YFP engagement with the β_2-adrenoceptor, which reached a peak between 4 and 6 min after addition of the agonist (Figure 7B). Thereafter, the BRET signal declined with time over the next 40 min (Figure 7). However, when 10 nM $VEGF_{165}a$ was added at the same time as isoprenaline, the activation of VEGFR2 altered the profile of the β_2-adrenoceptor engagement with β-arrestin2 (Figure 7B). Thus, the peak response was slightly truncated and thereafter a plateau was rapidly achieved (9 min after agonist addition), which was then maintained for the next 30 min (Figure 7B). This plateau remained significantly higher ($p < 0.05$) than that achieved with isoprenaline alone, where the response continued to decline. This effect of $VEGF_{165}a$ was completely prevented by pretreatment with the VEGFR2 RTK inhibitor cediranib (Figure 7B) (Carter et al., 2015). Studies of VEGFR2 phosphorylation using a phospho-specific antibody for the tyrosine residue 1,212 did not provide evidence for enhanced phosphorylation of VEGFR2 following stimulation with isoprenaline in cells co-expressing β_2-adrenoceptors (Figure S6). However, there was a significant degree of constitutive VEGFR2 phosphorylation in vehicle-treated cells (expressing both VEGFR2 and β_2-adrenoceptors) that was inhibited by cediranib.

DISCUSSION

Here, we have demonstrated that VEGFR2 and a GPCR (the β_2-adrenoceptor) functionally interact in highly specific hetero-oligomeric complexes to modulate receptor localization, trafficking, and downstream signaling. This was possible using NanoBRET technology, which sensitively detects interactions between protein-protein pairs (<10 nm apart). We found that the SNAP-tagged-β_2-adrenoceptor was in close proximity to NLuc-VEGFR2, indicative of complex formation. Notably, stimulation of either the GPCR or RTK with a selective agonist (β_2-adrenoceptor with isoprenaline or VEGFR2 receptor with $VEGF_{165}a$) was able to significantly enhance complex formation, as indicated by an increased BRET signal. In contrast, we found no evidence for interaction between the adenosine A_3 receptor and VEGFR2. This indicates that the RTK-GPCR heteromeric interactions observed here are not simply a consequence of non-specific bystander BRET, and that they are consistent with the documented physiological expression of β_2-adrenoceptors and VEGFR2, but not A_3 receptors, on endothelial cells (Claesson-Welsh and Welsh, 2013; Garg et al., 2017; Feoktistov et al.,

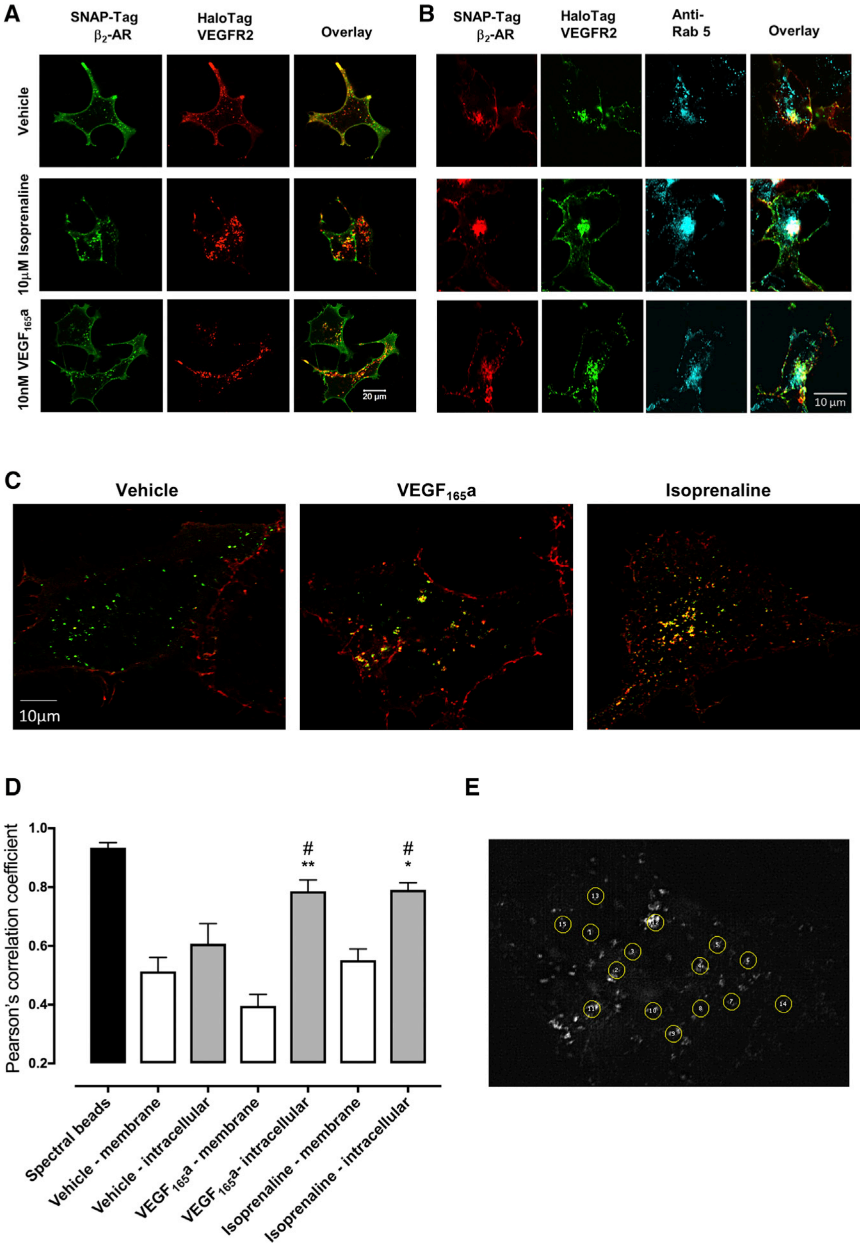

Figure 6. Influence of Agonists on the Cellular Location of Receptors and on Complex Formation between β_2-Adrenoceptors and β-Arrestin2

(A) Confocal imaging (Zeiss LSM 710) of HEK293 cells transiently co-transfected with 0.25 μg/well HaloTag- VEGFR2 and 0.25 μg/well SNAP-β_2-AR cDNAs, under unstimulated conditions (vehicle) or after treatment with 10 μM isoprenaline or 10 nM VEGF$_{165}$a ligands (30 min at 37°C). Data are representative of three individual experiments. Scale bar represents 20 μm.

(B) Immunolabeling of early endosomes (anti-Rab 5 antibody labeling). HEK293 cells transiently co-transfected with 0.5 μg/well HaloTag-VEGFR2 (green) and 0.5 μg/well SNAP-β_2-AR (red) cDNAs, under unstimulated conditions (vehicle) or after treatment with 10 μM isoprenaline or 10 nM VEGF$_{165}$a (30 min at 37°C). Cells were fixed using 3% paraformaldehyde/PBS, permeabilized using Triton X-100 (0.025% in PBS) and Rab 5 endosomal compartments labeled (cyan). Cells were imaged using a LSM880 confocal microscope (Zeiss). Data are representative of three individual experiments. Scale bar represents 10 μm.

(C) Structured illumination microscopy (SIM) super-resolution images of HEK293 cells transiently co-transfected with HaloTag-VEGFR2 (green) and SNAP-β_2-AR (red; 3 μg total cDNA). Cells were incubated with vehicle, 10 μM isoprenaline or 10 nM VEGF$_{165}$a (30 min at 37°C) before fixation and mounting onto microscope slides. Coverslips were imaged using a Zeiss ELYRA PS.1 microscope. Areas of co-localized HaloTag-VEGFR2 and SNAP-β_2-AR-labeled receptors are shown in yellow. Scale bar represents 10 μm.

(D and E) Summary of Pearson's correlation coefficients (D) obtained following co-localization analysis of SIM images of circular regions of interest (ROI) in HEK293 cells co-expressing HaloTag-VEGFR2 and SNAP-β_2-AR. ROI were placed on areas of fluorescence either at the plasma membrane or intracellular regions of SIM images of HEK293 cells co-expressing HaloTag-VEGFR2 (green; HaloTag AF488 membrane impermeant label) and SNAP-β_2-AR (red; SNAP AF647 membrane impermeant label). TetraSpeck microspheres (0.1-μm spectral beads stained with four fluorophores: 365/430 nm [blue], 505/515 nm [green], 560/580 nm [orange], and 660/680 nm [red]) were included in each experiment to allow X/Y/Z channel alignment correction in image processing. The Fiji (ImageJ) analysis program CoLoc2 was applied to these ROI (six ROIs for spectral bead images and 12–15 ROIs for all other conditions) and Pearson's correlation coefficients obtained. Values were averaged across all ROI and are expressed as means ± SEM. A Pearson correlation coefficient value of +1 implies a perfect co-occurrence of both green (HaloTag-VEGFR2) and red (SNAP-β_2-AR) fluorophores. *$p < 0.01$ or **$p < 0.001$ compared with equivalent membrane condition. #$p < 0.05$ compared with equivalent vehicle control. Examples of ROI are shown in (E).

2002). RTK-GPCR hetero-oligomerization was also demonstrated with genome-edited NLuc-β_2-adrenoceptor expressed under its endogenous promoter, where the extent of oligomerization was limited to physiological levels by the availability of the β_2-adrenoceptor. The level of exogenously expressed HaloTag-VEGFR2 that was available to interact with β_2-adrenoceptor in these cells was lower than endogenous levels of VEGFR2 in HUVECs (Peach et al., 2018b), supporting the physiological relevance of the observed hetero-oligomeric interactions.

These findings help to explain a striking feature of previous work that investigated the membrane-diffusional properties of receptor-ligand complexes in living cells using techniques such as fluorescence correlation spectroscopy (FCS) (Briddon et al., 2018). These studies suggested that the receptor species diffusing in the cell membrane were much larger than expected for a single membrane protein (or dimer) diffusing in isolation (Briddon et al., 2018). This suggests that these receptors may be normally present within macromolecular complexes of

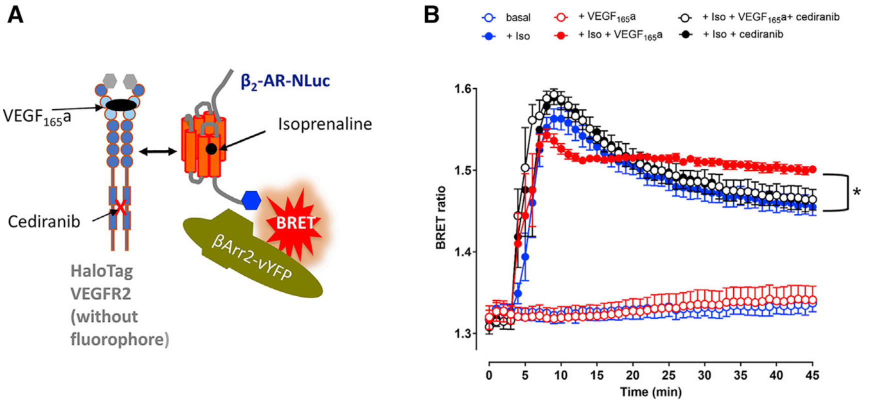

Figure 7. Influence of β_2-Adrenoceptor and VEGFR2 Co-expression on β-Arrestin2 Recruitment

(A) Scheme showing the experimental setup for the interaction between the β_2-adrenoceptor-NLuc (β_2-AR-NLuc) and β-arrestin2 (βArr2).

(B) βArr2 recruitment time course performed with HEK293 cells transiently co-transfected with 0.01 μg/well β_2-AR-NLuc, 0.04 μg/well β-arrestin2-Venus-YFP, and 0.04 μg/well HaloTag-VEGFR2. In these experiments the HaloTag substrate was not used and the HaloTag-VEGFR2 was used as an untagged construct. Cells were challenged with vehicle, 10 μM isoprenaline (Iso), or 10 μM isoprenaline plus 10 nM $VEGF_{165}a$ (all added at 4 min), in the presence or absence of 1 μM cediranib. *$p < 0.05$ between filled red and filled blue data points from 26 min onward (two-way ANOVA with repeated measures and Bonferroni's multiple comparison's tests). Data are means ± SEM from five (without cediranib treatment) or four (with cediranib treatment) separate experiments, each performed in triplicate wells.

much higher molecular mass and/or have restricted lateral diffusion. Since FCS has also identified multiple populations of ligand-receptor complexes for both GPCRs and RTKs, this suggests that multiple signaling complexes may exist in the plasma membrane and elsewhere (Briddon et al., 2018; Thomsen et al., 2016). These observations are consistent with recent work that has identified receptor-G protein interactions at cell surface protein hotspots (Sungkaworn et al., 2017; Calebiro and Sungkaworn, 2018) and also with work that has demonstrated continued intracellular signaling from GPCRs in endosomes (Irannejad et al., 2013; Eichel and von Zastrow, 2018), and the proposed importance of intracellular location to RTK signaling (Weddell and Imoukhuede, 2017).

Our work has also demonstrated that $VEGF_{165}a$-induced VEGFR2 homodimerization still occurs in the presence of β_2-adrenoceptor expression, suggesting that the oligomeric complexes detected by NanoBRET may be larger and contain more than just the two specific partner proteins under study. Thus, for example, $VEGF_{165}a$ may be enhancing the formation of oligomeric complexes containing multiple copies of both VEGFR2 and β_2-adrenoceptors since it can enhance the formation of both VEGFR2-VEGFR2 and VEGFR2-β_2-adrenoceptor complexes (as determined by NanoBRET). In addition, β_2-adrenoceptors homodimers appear to be preformed since their formation is not influenced by agonist (isoprenaline or $VEGF_{165}a$) stimulation.

The studies described here also provide evidence that receptor oligomerization can modulate the localization and trafficking of receptors. Confocal imaging studies in living cells expressing an N-terminal SNAP-tagged β_2-adrenoceptor and an N-terminal-HaloTag variant of VEGFR2 showed that β_2-adrenoceptors largely remained on the plasma membrane under basal conditions, whereas VEGFR2 (as observed by us previously [Kilpatrick et al., 2017]) underwent constitutive internalization. When β_2-adrenoceptors and VEGFR2 were expressed together, there was enhanced constitutive internalization of the β_2-adrenoceptor and some co-localization with VEGFR2 at intracellular sites. This was markedly enhanced following treatment with isoprenaline and co-localized receptors were observed in Rab5-positive intracellular endosomes. A similar situation occurred following $VEGF_{165}a$ stimulation. Collectively these data suggest that, following agonist stimulation of one of the receptor partners, there is enhanced co-localization (Figure 6) and oligomerization (Figure 3) between VEGFR2 and β_2-adrenoceptors within intracellular endosomes.

The findings suggest that receptor hetero-oligomerization may have evolved to expand the scope of downstream signaling that occurs in response to receptor agonism. NanoBRET assays demonstrated that the β_2-adrenoceptor can regularly engage with both a surrogate of the Gs-alpha subunit (Nb80) and with β-arrestin2.

In the case of β-arrestin2, interaction of β_2-adrenoceptors occurred rapidly (within 4–6 min), with a gradual decline in complex formation (indicated by reduced NanoBRET signal), indicating dissociation of the receptor and signaling molecule. While VEGFR2 stimulation with $VEGF_{165}a$ had no effect on the absolute ability of the β_2-adrenoceptor to engage with downstream signaling molecules, it was notable that simultaneous stimulation of co-expressed β_2-adrenoceptors and VEGFR2 altered the temporal characteristics of the interaction between the β_2-adrenoceptor and β-arrestin2. Thus, the maximal level of β_2-adrenoceptor/β-arrestin2 complex formation was truncated, but the rate of complex dissociation was reduced, resulting in sustained complexes for the duration of the experiment (>30 min). The requirement for VEGFR2 activation to alter β_2-adrenoceptor signaling dynamics was confirmed by blocking the effect of $VEGF_{165}a$ by the VEGFR2 RTK inhibitor, cediranib (Carter et al., 2015). Since activation of both VEGFR2 and β_2-adrenoceptor drive internalization of receptor complexes, it is plausible that sustained β_2-adrenoceptor/β-arrestin2 signaling occurs in intracellular endosomes, which may have particular implications for cellular proliferation and adds further complexity to the spatial-temporal control of β_2-adrenoceptor signaling (Irannejad et al., 2013; Eichel and von Zastrow, 2018). These observations may be particularly relevant to the observed attenuation of VEGF-induced cellular proliferation in HUVECs when concentrations of β_2-agonist are used that will stimulate the recruitment of β-arrestin2 to the receptor (Figure 7). Thus, in the presence of 10 μM isoprenaline, the effect of 3 nM $VEGF_{165}a$ on cell proliferation is substantially reduced in human endothelial cells (Figure S2) and this may be related to altered signaling from intracellular endosomes. High concentrations of isoprenaline

(5–10 μM) have also been shown to enhance VEGF-dependent angiogenic sprouting in HUVECs (Garg et al., 2017). Consistent with this interaction between VEGFR2 and β_2-adrenoceptors in physiologically relevant human endothelial cells, we have also obtained evidence for VEGFR2-β_2-adrenoceptor oligomerization (detected by BRET) in HUVECs.

The NanoBRET technology used in the current work represents a powerful proximity-based methodology to monitor protein-protein interactions involving different receptors in living cells, as well as ligand binding to cell surface receptors. It reports on the close proximity between two tagged partners that are localized within 10 nm of each other (Stoddart et al., 2018). The NanoBRET methodology also allowed us to investigate if cooperativity occurred across interfaces of the oligomeric complex of VEGFR2 with the β_2-adrenoceptor, and demonstrated that no such cooperativity occurs. We have previously shown, using NanoBRET, that agonist-occupied receptor complexes are rapidly internalized to intracellular endosomes (Kilpatrick et al., 2017), and imaging studies in the current study suggest that this is associated with internalization of VEGFR2-β_2-adrenoceptor oligomeric complexes. Furthermore, NanoBRET studies demonstrated that these receptor oligomers can associate with, and alter signaling via, β-arrestin2.

In summary, we have demonstrated here that oligomeric complexes involving VEGFR2 and β_2-adrenoceptors can be generated in cell membranes and intracellular endosomes that retain their ability to couple to intracellular signaling pathways. Complexes involving the β_2-adrenoceptor and VEGFR2 can co-internalize following agonist treatment. Furthermore, $VEGF_{165}$a or isoprenaline can stimulate further complex formation. $VEGF_{165}$a treatment can also alter the temporal characteristics of the isoprenaline-stimulated association between the β_2-adrenoceptor and β-arrestin2. These data suggest that VEGFR2 and the β_2-adrenoceptor may be key components of membrane and endosomal macromolecular complexes, which may have substantial implications for the spatiotemporal control of signaling driven by both of these receptors in physiological or pathophysiological states. Furthermore the formation of VEGFR2/β_2-adrenoceptor complexes may provide insights into their combined effects on endothelial cell proliferation and the therapeutic benefit of the β_2-adrenoceptor antagonist propranolol in the treatment of infantile hemangioma. In addition, as both RTK and GPCR family members have been implicated in driving cancer progression and metastasis, the potential formation of distinct RTK/GPCR complexes may represent anti-cancer therapeutic targets for drug discovery efforts to exploit.

SIGNIFICANCE

The vascular endothelial growth factor receptor 2 (VEGFR2) is a key mediator of angiogenesis and endothelial cell proliferation following binding of its cognate ligand VEGF. Therefore the VEGFR2/VEGF signaling axis is an attractive therapeutic target in the treatment of conditions characterized by aberrant angiogenesis, such as cancer, and the common childhood tumor, infantile hemangioma. Interestingly the β-adrenoceptor antagonist propranolol is the current first-line treatment for infantile hemangioma; however, its mechanism of action is not fully understood, although it is believed to ultimately result in decreased VEGF expression and cell proliferation. Activation of the β_2-adrenoceptor has also been suggested to augment VEGFR2 signaling in multiple cancer types. We have used bioluminescence resonance energy transfer (BRET) to demonstrate the existence of oligomeric complexes between VEGFR2 and the β_2-adrenoceptor at the plasma membrane and also within intracellular compartments of living HEK293 cells and human umbilical vein endothelial cells (HUVECs). The use of CRISPR/Cas9 gene editing in HEK293 cells illustrated that VEGFR2/β_2-adrenoceptor complexes were present, even when the β_2-adrenoceptor was expressed at endogenous levels. These complexes can be induced by either VEGFR2 or β_2-adrenoceptor selective ligands, and are able to co-internalize to shared intracellular compartments. These oligomeric complexes can couple to intracellular signaling proteins, and the presence of activated VEGFR2 is able to alter the temporal profile of β-arrestin2 coupling to the β_2-adrenoceptor in response to ligand stimulation. The existence of VEGFR2/β_2-adrenoceptor complexes may explain why the β_2-adrenoceptor-selective antagonist, propranolol, is therapeutically beneficial in the treatment of infantile hemangioma. In addition the documented synergy of VEGFR2 and β_2-adrenoceptor signaling in many cancer types suggests that the formation of complexes between these two receptors subtypes could represent anti-cancer therapeutic targets.

STAR★METHODS

Detailed methods are provided in the online version of this paper and include the following:

- KEY RESOURCES TABLE
- CONTACT FOR REAGENT AND RESOURCE SHARING
- EXPERIMENTAL MODEL AND SUBJECT DETAILS
- METHOD DETAILS
  - Materials
  - Molecular Biology
  - CRISPR/Cas9 Guide and Donor Vector Cloning
  - Cell Culture
  - NanoBRET Assays to Determine Fluorescent Ligand Saturation Binding
  - NanoBRET Saturation Assays to Investigate Receptor-Receptor Interactions
  - Widefield Bioluminescence Microscopy
  - NanoBRET Assays to Investigate the Effects of Isoprenaline or $VEGF_{165}$a on Heteromer Formation
  - NanoBRET Assays to Investigate Potential for Cooperativity across Putative Receptor Dimer Interfaces
  - VEGFR2 Phosphorylation Assay
  - HUVEC Proliferation Assay
  - NFAT Luciferase Reporter Gene Assay
  - β-arrestin Recruitment Assays
  - Nanobody-80 Recruitment Assays
  - Live Cell Confocal Imaging
  - Rab5 Immunolabelling
  - Structured Illumination Microscopy

- QUANTIFICATION AND STATISTICAL ANALYSIS
- DATA AND SOFTWARE AVAILABILITY

SUPPLEMENTAL INFORMATION

Supplemental Information can be found online at https://doi.org/10.1016/j.chembiol.2019.02.014.

ACKNOWLEDGMENTS

This work was funded by BBSRC (grant number BB/L019418/1); the MRC (grant number MR/N020081/1); and Promega Corporation. Super-resolution microscopy was performed at the Multidisciplinary Super Resolution Microscopy Facility at the University of Nottingham, and funded by the BBSRC (grant number BB/L013827/1). E.K.S. is supported by NHMRC project grant 1147498. K.D.G.P. is funded by an NHMRC RD Wright Fellowship (1085842) and C.W.W. by an NHMRC CJ Martin Fellowship (1088334). K.D.G.P. is a Chief Investigator, and K.V.W. and S.J.H. are Partner Investigators of ARC Linkage Grant LP160100857, which includes associated financial support from Promega Corporation, BMG Labtech, and Dimerix. S.J.H. and L.E.K. thank the Raine Foundation for a Visiting Research Professorship and The University of Western Australia for a Research Collaboration Award, respectively, to visit the University of Western Australia.

AUTHOR CONTRIBUTIONS

S.J.H., J.W., and L.E.K. conceived the study. S.J.H., J.W., L.E.K., and E.K.S. supervised the project. R.F.O., K.Z., A.K., K.D.G.P., and C.W.W. generated reagents. L.E.K., D.C.A., S.J.H., and J.W. participated in research design. L.E.K., D.C.A., C.W.W., and C.J.P. conducted the experiments. L.E.K., D.C.A., S.J.H., and C.W.W. performed the data analysis. L.E.K., D.C.A., C.W.W., K.D.G.P., K.V.W., M.B.R., R.F.O., E.K.S., J.W., and S.J.H. wrote or contributed to the writing of the manuscript.

DECLARATIONS OF INTERESTS

R.F.O., M.B.R., K.Z., and K.V.W. are employees of Promega Corporation, which has proprietary rights over the NanoBRET assay and HaloTag technology. K.D.G.P. is Chief Scientific Adviser of Dimerix, a spin-out company of The University of Western Australia that has been assigned the rights to the Receptor Heteromer Investigation Technology. K.D.G.P. is an inventor on patents covering the technology and has a shareholding in Dimerix.

Received: September 21, 2018
Revised: December 30, 2018
Accepted: February 24, 2019
Published: April 4, 2019

REFERENCES

Baker, J.G., Hall, I.P., and Hill, S.J. (2003). Pharmacology and direct visualisation of BODIPY-TMR-CGP: a long acting fluorescent β_2-adrenoceptor agonist. Br. J. Pharmacol. *139*, 232–242.

Bergelin, N., Löf, C., Balthasar, S., Kalhori, V., and Törnquist, K. (2010). S1P1 and VEGFR-2 form a signaling complex with extracellularly regulated kinase 1/2 and protein kinase C-alpha regulating ML-1 thyroid carcinoma cell migration. Endocrinology *151*, 2994–3005.

Bessman, N.J., Bagchi, A., Ferguson, K.M., and Lemmon, M.A. (2014a). Complex relationship between ligand binding and dimerization in the epidermal growth factor receptor. Cell Rep. *9*, 1306–1317.

Bessman, N.J., Freed, D.M., and Lemmon, M.A. (2014b). Putting together structures of epidermal growth factor receptors. Curr. Opin. Struct. Biol. *29*, 95–101.

Briddon, S.J., Kilpatrick, L.E., and Hill, S.J. (2018). Studying GPCR pharmacology in membrane microdomains: fluorescence correlation spectroscopy comes of age. Trends Pharmacol. Sci. *39*, 158–174.

Brozzo, M.S., Bjelic, S., Kisko, K., Schleier, T., Peppanen, V.M., Alitalo, K., Winkler, F.M., and Ballmer-Hofer, K. (2012). Thermodynamic and structural description of allosterically regulated VEGFR-2 dimerization. Blood *119*, 1781–1788.

Calebiro, D., and Sungkaworn, T. (2018). Single molecule imaging of GPCR interactions. Trends Pharmacol. Sci. *39*, 109–122.

Carter, J.J., Wheal, A.J., Hill, S.J., and Woolard, J. (2015). Effects of receptor tyrosine kinase inhibitors on $VEGF_{165}$a- and $VEGF_{165}$b-stimulated NFAT-mediated gene transcription in HEK-293 cells expressing the human vascular endothelial growth factor receptor 2 (VEGFR2). Br. J. Pharmacol. *172*, 3141–3150.

Chang, A., Le, C.P., Walker, A.K., Creed, S.J., Pon, C.K., Albold, S., Carrol, D., Halls, M.L., Lane, J.R., Riedel, B., et al. (2016). β2-Adrenoceptors on tumor cells play a critical role in stress-enhanced metastasis in a mouse model of breast cancer. Brain Behav. Immun. *57*, 106–115.

Claesson-Welsh, L., and Welsh, M. (2013). VEGFA and tumour angiogenesis. J. Int. Med. *273*, 114–127.

Daub, H., Wallasch, C., Lankenau, A., Herrlich, A., and Ullrich, A. (1997). Signal characteristics of G protein-transactivated EGF receptor. EMBO J. *16*, 7032–7044.

Dixon, J.E., Dick, E., Rajamohan, D., Shakesheff, K.M., and Denning, C. (2011). Directed differentiation of human embryonic stem cells to interrogate the cardiac gene regulatory network. Mol. Ther. *19*, 1695–1703.

Dosch, D.D., and Ballmer-Hofer, K. (2010). Transmembrane domain-mediated orientation of receptor monomers in active VEGFR-2 dimers. FASEB J. *24*, 32–38.

Eichel, K., and von Zastrow, M. (2018). Subcellular organization of GPCR signalling. Trends Pharmacol. Sci. *39*, 200–208.

Feoktistov, I., Goldstein, A.E., Ryzhov, S., Zeng, D., Belardinelli, L., Voyno-Yasenetskaya, T., and Biaggioni, I. (2002). Differential expression of adenosine receptors in human endothelial cells: role of A2B receptors in angiogenic factor regulation. Circ. Res. *90*, 531–538.

Ferrara, N. (2009). VEGF-A: a critical regulator of blood vessel growth. Eur. Cytokine Netw. *20*, 158–163.

Ferré, S., Casadó, V., Devi, L.A., Filizola, M., Jockers, R., Lohse, M.J., Milligan, G., Pin, J.P., and Guitart, X. (2014). G protein–coupled receptor oligomerization revisited: functional and pharmacological perspectives. Pharmacol. Rev. *66*, 413–434.

Freed, D.M., Alvarado, D., and Lemmon, M.A. (2015). Ligand regulation of a constitutively dimeric EGF receptor. Nat. Commun. *6*, 7380.

Garg, J., Feng, Y.X., Jansen, S.R., Friedrich, J., Lezoualc'h, F., Schmidt, M., and Weiland, T. (2017). Catecholamines facilitate VEGF-dependent angiogenesis via β_2-adrenoceptor-induced Epac1 and PKA activation. Oncotarget *8*, 44732–44748.

George, A.J., Hannan, R.D., and Thomas, W.G. (2013). Unravelling the molecular complexity of GPCR-mediated EGFR transactivation using functional genomics approaches. FEBS J. *280*, 5258–5268.

Gherbi, K., May, L.T., Baker, J.G., Briddon, S.J., and Hill, S.J. (2015). Negative cooperativity across β_1-adrenoceptor homodimers provides insights into the nature of the secondary low affinity "CGP 12177" β_1-adrenoceptor binding conformation. FASEB J. *29*, 2859–2871.

Gibson, D.G., Young, L., Chuang, R.Y., Venter, J.C., Hutchison, C.A., and Smith, H.O. (2009). Enzymatic assembly of DNA molecules up to several hundred kilobases. Nat. Methods *6*, 343–345.

Hart, S., Fischer, O.M., Prenzel, N., Zwick-Wallasch, E., Schneider, M., Hennighausen, L., and Ullrich, A. (2005). GPCR-induced migration of breast carcinoma cells depends on both EGFR signal transactivation and EGFR-independent pathways. J. Biol. Chem. *386*, 845–855.

Hsu, P.D., Scott, D.A., Weinstein, J.A., Ran, F.A., Konermann, S., Agarwala, V., Li, Y., Fine, E.J., Wu, X., et al. (2013). DNA targeting specificity of RNA-guided Cas9 nucleases. Nat. Biotechnol. *31*, 827–832.

Hulsurkar, M., Li, Z., Zhang, Y., Li, X., Zheng, D., and Li, W. (2017). Beta-adrenergic signaling promotes tumor angiogenesis and prostate cancer progression through HDAC2-mediated suppression of thrombospondin-1. Oncogene *36*, 1525–1536.

Irannejad, R., Tomshine, J.C., Tomshine, J.R., Chevalier, M., Mahoney, J.P., Steyaert, J., Rasmussen, S.G., Sunahara, R.K., El-Samad, H., Hunag, B., and von Zastrow, M. (2013). Conformational biosensors reveal GPCR signalling from endosomes. Nature *495*, 534–538.

Jaeger, W.C., Armstrong, S.P., Hill, S.J., and Pfleger, K.D.G. (2014). Biophysical detection of diversity and bias in GPCR function. Front. Endocrinol. (Lausanne) *5*, 26.

Kent, W.J., Sugnet, C.W., Furey, T.S., Roskin, K.M., Pringle, T.H., Zahler, A.M., and Haussler, D. (2002). The human genome browser at UCSC. Genome Res. *12*, 996–1006.

Kilpatrick, L.E., Friedman-Ohana, R., Alcobia, D.C., Riching, K., Peach, C.J., Wheal, A.J., Briddon, S.J., Robers, M.B., Zimmerman, K., Machleidt, T., et al. (2017). Real-time analysis of the binding of fluorescent VEGF165a to VEGFR2 in living cells: effect of receptor tyrosine kinase inhibitors and fate of internalized agonist-receptor complexes. Biochem. Pharmacol. *136*, 62–75.

Kocan, M., See, H.B., Seeber, R.M., Eidne, K.A., and Pfleger, K.D. (2008). Demonstration of improvements to the bioluminescence resonance energy transfer (BRET) technology for the monitoring of G protein-coupled receptors in live cells. J. Biomol. Screen *13*, 888–898.

Laporte, S.A., Oakley, R.H., Holt, J.A., Barak, L.S., and Caron, M.G. (2000). The interaction of β-arrestin with the AP-2 adaptor is required for the clustering of β2-adrenergic receptor into clathrin-coated pits. J. Biol. Chem. *275*, 23120–23126.

Léauté-Labrèze, C., Dumas del la Roque, E., Hubiche, T., Boralevi, F., Thambo, J.B., and Taieb, A. (2008). Propranolol for severe hemangiomas of infancy. New Eng. J. Med. *358*, 2649–2651.

Liebmann, C. (2011). EGF receptor activation by GPCRs: an universal pathway reveals different versions. Mol. Cell. Endocrinol. *331*, 222–231.

Luttrell, L.M., Daaka, Y., and Lefkowitz, R.J. (1999). Regulation of tyrosine kinase cascades by G-protein-coupled receptors. Curr. Opin. Cell Biol. *11*, 177–183.

Luttrell, L.M., Roudabush, F.L., Choy, E.W., Miller, W.E., Field, M.E., Pierce, K.L., and Lefkowitz, R.J. (2001). Activation and targeting of extracellular signal-regulated kinases by β-arrestin scaffolds. Proc. Natl. Acad. Sci. U S A *98*, 2449–2454.

Markovic-Mueller, S., Stuttfeld, E., Asthana, M., Weinert, T., Bliven, S., Goldie, K.N., Kisko, K., Capitani, G., and Ballmer-Hofer, K. (2017). Structure of the full-length VEGFR-1 extracellular domain in complex with VEGF-A. Structure *25*, 341–352.

May, L.T., Bridge, L.J., Stoddart, L.A., Briddon, S.J., and Hill, S.J. (2011). Allosteric interactions across native adenosine-A_3 receptor homodimers: quantification using single cell ligand binding kinetics. FASEB J. *25*, 3465–3476.

Mercier, J.F., Salahpour, A., Angers, S., Breit, A., and Bouvier, M. (2002). Quantitative assessment of β1- and β2-adrenergic receptor homo- and heterodimerization by bioluminescence resonance energy transfer. J. Biol. Chem. *277*, 44925–44931.

Mulcrone, P.L., Campbell, J.P., Clément-Demange, L., Anbinder, A.L., Merkel, A.R., Brekken, R.A., Sterling, J.A., and Elefteriou, F. (2017). Skeletal colonization by breast cancer cells is stimulated by an osteoblast and β2AR-dependent neo-angiogenic switch. J. Bone Miner. Res. *32*, 1442–1454.

Musumeci, F., Radi, M., Brullo, C., and Schenone, S. (2012). Vascular endothelial growth factor (VEGF) receptors: drugs and new inhibitors. J. Med. Chem. *55*, 10797–10822.

Ou, J.M., Yu, Z.Y., Qiu, M.K., Dai, Y.X., Dong, Q., Shen, J., Wang, X.F., Liu, Y.B., Quan, Z.W., and Fei, Z.W. (2014). Knockdown of VEGFR2 inhibits proliferation and induces apoptosis in hemangioma derived endothelial cells. Eur. J. Histochem. *58*, 2263.

Ozeki, M., Nozawa, A., Hori, T., Kanda, K., Kimura, T., Kawamoto, N., and Fukao, T. (2016). Propranolol for infantile hemangioma: effect on plasma vascular endothelial growth factor. Pediatr. Int. *58*, 1130–1135.

Park, S.Y., Kang, J.H., Jeong, K.J., Lee, J., Han, J.W., Choi, W.S., Kim, Y.K., Kang, J., Park, C.G., and Lee, H.Y. (2011). Norepinephrine induces VEGF expression and angiogenesis by a hypoxia-inducible factor-1α protein-dependent mechanism. Int. J. Cancer *128*, 2306–2316.

Parmar, V.K., Grinde, E., Mazurkiewicz, J.E., and Herrick-Davis, K. (2016). Beta2-adrenergic receptor homodimers: role of transmembrane domain 1 and helix 8 in dimerization and cell surface expression. Biochim. Biophys. Acta *1859*, 1445–1455.

Peach, C.J., Mignone, V.W., Arruda, M.A., Alcobia, D.C., Hill, S.J., Kilpatrick, L.E., and Woolard, J. (2018a). Molecular pharmacology of VEGF-A isoforms: binding and signalling at VEGFR2. Int. J. Mol. Sci. *19*, https://doi.org/10.3390/ijms19041264.

Peach, C.J., Kilpatrick, L.E., Friedman-Ohana, R., Zimmerman, K., Robers, M.B., Wood, K.V., Woolard, J., and Hill, S.J. (2018b). Real-time ligand binding of fluorescent VEGF-A isoforms that discriminate between VEGFR2 and NRP1 in living cells. Cell Chem. Biol. *25*, 1208–1218.

Prenzel, N., Zwick, E., Daub, H., Leserer, M., Abraham, R., Wallasch, C., and Ullrich, A. (1999). EGF receptor transactivation by G-protein-coupled receptors requires metalloproteinase cleavage of proHB-EGF. Nature *402*, 884–888.

Pyne, N.J., and Pyne, S. (2011). Receptor tyrosine kinase-G-protein-coupled receptor signalling platforms: out of the shadow? Trends Pharmacol. Sci. *32*, 443–450.

Ran, F.A., Hsu, P.D., Wright, J., Agarwala, V., Scott, D.A., and Zhang, F. (2013). Genome engineering using the CRISPR-Cas9 system. Nat. Protoc. *8*, 2281–2308.

Rasmussen, S.G., Choi, H.J., Fung, J.J., Pardon, E., Casarosa, P., Chae, P.S., Devree, B.T., Rosenbaum, D.M., Thian, F.S., Kobilka, T.S., et al. (2011). Structure of a nanobody-stabilized active state of the β_2-adrenoceptor. Nature *469*, 175–180.

Sarabipour, S., Ballmer-Holfer, K., and Hritova, K. (2016). VEGFR2 conformational switch in response to ligand binding. Elife *5*, e13876.

Shenoy, S.K., and Lefkowitz, R.J. (2011). β-Arrestin-mediated receptor trafficking and signal transduction. Trends Pharmacol. Sci. *32*, 521–533.

Shibuya, M. (2011). Vascular endothelial growth factor (VEGF) and its receptor (VEGFR) signaling in angiogenesis: a crucial target for anti- and pro-angiogenic therapies. Genes Cancer *2*, 1097–1105.

Sloan, E.K., Priceman, S.J., Cox, B.F., Yu, S., Pimentel, M.A., Tangkanangnukul, V., Arevalo, J.M., Morizono, K., Karanikolas, B.D., Wu, L., et al. (2010). The sympathetic nervous system induces a metastatic switch in primary breast cancer. Cancer Res. *70*, 7042–7052.

Steyaert, J., and Kobilka, B.K. (2011). Nanobody stabilization of G protein-coupled receptor conformational states. Curr. Opin. Struct. Biol. *21*, 567–572.

Stiles, J., Amaya, C., Pham, R., Rowntree, R.K., Lacaze, M., Mulne, A., Bischoff, J., Kokta, V., Boucheron, L.E., Mitchell, D.C., and Bryan, B.A. (2012). Propranolol treatment of infantile hemangioma endothelial cells: a molecular analysis. Exp. Ther. Med. *4*, 594–604.

Stoddart, L.A., Kellam, B., Briddon, S.J., and Hill, S.J. (2014). Effect of a toggle switch mutation in TM6 of the human adenosine A3 receptor on Gi protein-dependent signalling and Gi-independent receptor internalization. Br. J. Pharmacol. *171*, 3827–3844.

Stoddart, L.A., Johnstone, E.K.M., Wheal, A.J., Goulding, J., Robers, M.B., Machleidt, T., Wood, K.V., Hill, S.J., and Pfleger, K.D.G. (2015). Application of BRET to monitor ligand binding to GPCRs. Nat. Methods *12*, 661–663.

Stoddart, L.A., Kilpatrick, L.E., and Hill, S.J. (2018). NanoBRET approaches to study ligand binding to GPCRs and RTKs. Trends Pharmacol. Sci. *39*, 136–147.

Sungkaworn, T., Jobin, M.L., Burnecki, K., Weron, A., Lohse, M.J., and Calebiro, D. (2017). Single-molecule imaging reveals receptor-G protein interactions at cell surface hot spots. Nature *550*, 543–547.

Thomsen, A.R.B., Plouffe, B., Cahill, T.J., III, Shukla, A.K., Tarrasch, J.T., Dosey, A.M., Kahsai, A.W., Strachan, R.T., Pani, B., Mahoney, J.P., et al. (2016). GPCR-G protein-β-arrestin super-complex mediates sustained G protein signalling. Cell *166*, 907–919.

Thuringer, D., Maulon, L., and Frelin, C. (2002). Rapid transactivation of the vascular endothelial growth factor receptor KDR/Flk-1 by the bradykinin B2 receptor contributes to endothelial nitric-oxide synthase activation in cardiac capillary endothelial cells. J. Biol. Chem. *277*, 2028–2032.

Vischer, H.F., Castro, M., and Pin, J.P. (2015). G protein-coupled receptor multimers: a question still open despite the use of novel approaches. Mol. Pharmacol. *88*, 561–571.

Weddell, J.C., and Imoukhuede, P.I. (2017). Integrative meta-modelling identifies endocytic vesicle, late endosome and the nucleus as the cellular compartments primarily directing RTK signaling. Integr. Biol. (Camb) *9*, 464–484.

White, C.W., Vanyai, H.K., See, H.B., Johnstone, E.K.M., and Pfleger, K.D.G. (2017). Using nanoBRET and CRISPR/Cas9 to monitor proximity to a genome-edited protein in real time. Sci. Rep. *7*, 3187.

Zajac, M., Law, J., Cvetkovic, D.D., Pampillo, M., McColl, L., Pape, C., Di Guglielmo, G.M., Postovit, L.M., Babwah, A.V., and Bhattacharya, M. (2011). GPR54 (KISS1R) transactivates EGFR to promote breast cancer cell invasiveness. PLoS One *6*, e21599.

STAR★METHODS

KEY RESOURCES TABLE

REAGENT or RESOURCE	SOURCE	IDENTIFIER
Antibodies		
Anti Rab5 antibody (rabbit monoclonal; primary)	New England Biolabs (Cell Signaling Technology)	Cat# 3547; RRID: AB_2300649
Donkey anti rabbit IgG Alexa Fluor 568 (secondary)	ThermoFisher Scientific, USA	Cat# A10042; RRID: AB_2534017
Rabbit monoclonal anti-VEGFR2 phosphoY1212	Cell Signalling	Cat# 2477; RRID: AB_331374
Chicken anti rabbit AF488 conjugated secondary antibody	Thermo Fisher Scientfic, USA	Cat# A21441; RRID: AB_141735
Chemicals, Peptides, and Recombinant Proteins		
$VEGF_{165}a$	R&D Systems (Abingdon, UK)	Cat# 293-VE
$VEGF_{165}a$-TMR	Promega Corporation (Wisconsin, USA)	Custom synthesis
HaloTag AlexaFluor 488 membrane impermeant substrate	Promega Corporation (Wisconsin, USA)	Cat# G1002
HaloTag AlexaFluor 660 membrane impermeant substrate	Promega Corporation (Wisconsin, USA)	Cat# G8471
SNAPTag AlexaFluor 488 membrane impermeant substrate	New England Biolabs	Cat# S9124S
SNAPTag AlexaFluor 647 membrane impermeant substrate	New England Biolabs	Cat# S9136S
Formaldehyde solution 4%	Sigma Aldrich	Cat# F8775
Cediranib	Sequoia Research Products	Cat# SRP01883c
Protease-free bovine serum albumin	Sigma Aldrich	Cat# 03117332001
ProLong Glass antifade reagent	ThermoFisher Scientific, USA	Cat# P36965
Dulbecco's Modified Eagle's Medium	Sigma Aldrich	Cat# D6429
CitiFluor mounting medium	CitiFluor, USA	Cat# E17979-20
Medium 200	ThermoFisher Scientific, USA	Cat# M200500
LVES 50x (large vessel endothelial cell supplement)	ThermoFisher Scientific, USA	Cat# A14608-01
Immersol^{TM} 518F (30°C) oil	Zeiss, Germany	Cat# 444970-9000-000
Ingenio electroporation kit	Mirus Bio	Cat# MIR50114
Fetal Bovine Serum	Sigma Aldrich	Cat# F2442
Propranolol hydrochloride	Tocris	Cat# 0624
Isoproterenol (isoprenaline) hydrochloride	Sigma Aldrich	Cat# I6504
ICI 118551 hydrochloride	Tocris	Cat# 0821
CGP12177 hydrochloride	Tocris	Cat# 1134
CGP12177-TMR	Molecular Probes, Oregon, USA	Described in Baker et al., 2003
Opti-MEM reduced serum medium	ThermoFisher Scientific	Cat# 11058021
Poly-D-Lysine hydrobromide Dulbecco's	Sigma Aldrich	Cat# P6407
Dulbecco's Phosphate Buffered Saline (DPBS)	Sigma Aldrich	Cat# D8537
Trypsin-EDTA solution x10	Sigma Aldrich	Cat# T4174
Chicken serum	Sigma Aldrich	Cat# C5405
Puromycin dihydrochloride from Streptomyces alboniger	Sigma Aldrich	Cat# P8833
FuGENE HD	Promega	Cat# E2311
Triton-X-100	Sigma Aldrich	Cat# X100
Glycine	Sigman Aldrich	Cat# G8898
bisBenzimide H33342 trihydrochloride	Sigma Aldrich	Cat# B2261
Critical Commercial Assays		
ONE-Glo^{TM} Luciferase	Promega Corporation (Wisconsin, USA)	Cat# E6120
Nano-Glo luciferase assay system (Furimazine)	Promega Corporation (Wisconsin,USA)	Cat# N1130

(*Continued on next page*)

Continued

REAGENT or RESOURCE	SOURCE	IDENTIFIER
Experimental Models: Cell Lines		
Human: GloResponse™ NFAT-RE-luc2P HEK293 cell line (female)	Promega Corporation (Wisconsin, USA)	Cat# E8510
Human: HEK293T cells (female)	ATCC (Virginia, USA)	Cat# CRL-3216
Human: HUVEC cells (newborn male, single donor)	ThermoFisher Scientific	Cat# C0035C. Lot number: 1606186.
Recombinant DNA		
NanoLuc-VEGFR2	Promega Corporation (Wisconsin, USA)	Custom synthesis
HaloTag-VEGFR2	Promega Corporation (Wisconsin, USA)	Custom synthesis
NanoLuc-β_2AR	Promega Corporation (Wisconsin, USA)	Custom synthesis
NanoLuc-A_3R	Stoddart et al., 2015	Custom synthesis
SnapTag-β_2AR	Gherbi et al., 2015	Custom synthesis
SnapTag- A_3R	Stoddart et al., 2014	Custom synthesis
pSIN-Nb-80-GFP	Described in this manuscript.	Custom synthesis
β_2AR-NanoLuc	Promega Corporation (Wisconsin, USA)	Custom synthesis
β-arrestin2-Venus-YFP	Kocan et al., 2008	Custom synthesis
pSpCas9(BB)-2A-Puro (PX459) V2.0	Addgene	Plasmid #62988; RRID: Addgene_62988
ADRB2 Homology directed repair template	GeneArt (Thermofisher Scientific)	Custom synthesis
Oligonucleotides	Sigma Aldrich	Custom synthesis
Software and Algorithms		
GraphPad Prism 7.02	GraphPad Software, La Jolla California USA	https://www.graphpad.com/scientific-software/prism/
Zen 2010	Zeiss, Germany	www.zeiss.com
ImageJ Fiji 1.52e (Coloc2 plug in)	National Institute of Health, USA	https:/fiji.sc
Other		
White 96-well plates	Greiner Bio-One	Cat# 655089
Black 96-well plates	Greiner Bio-One	Cat# 655090
Nunc Lab-Tek 8-well chambered coverslips	ThermoFisher Scientific	Cat# 1554411
Coverslips (18x18mm; 1.5H)	Zeiss, Germany	Cat# 474030-9000-000
4-chamber 35mm dish with 20mm bottom well containing 1.5μm glass coverslip	Cellvis, California, USA	Cat# D35C4-20-1.5-N
BbsI restriction enzyme	New England Biolabs (UK)	Cat# R0539S
BamH1 restriction enzyme	Promega Corporation (Wisconsin, USA)	Cat# R6021
BglII restriction enzyme	Promega Corporation (Wisconsin, USA)	Cat# R6081
XhoI restriction enzyme	Promega Corporation (Wisconsin, USA)	Cat# R6161
XbaI restriction enzyme	Promega Corporation (Wisconsin, USA)	Cat# R6181
KpnI restriction enzyme	Promega Corporation (Wisconsin, USA)	Cat# R6341
SpeI restriction enzyme	Promega Corporation (Wisconsin, USA)	Cat# R6591
T4 DNA ligase	Promega Corporation (Wisconsin, USA)	Cat# M1801
Gibson Assemby Master mix	New England Biolabs (UK)	Cat# E26115
Pfu DNA polymerase	Promega Corporation (Wisconsin, USA)	Cat# 7741
TetraSpeck™ microspheres (0.1μm)	Thermo Fisher Scientific	Cat# T7279

CONTACT FOR REAGENT AND RESOURCE SHARING

Further information and requests for resources and reagents should be directed to and will be fulfilled by the Lead Contact, Stephen J. Hill (stephen.hill@nottingham.ac.uk).

EXPERIMENTAL MODEL AND SUBJECT DETAILS

Human HEK293T cells (female) were obtained from ATCC (Virginia, USA) and the human GloResponse™ NFAT-RE-luc2P HEK293 cell line (female) was obtained from Promega Corporation (Wisconsin, USA). Human umbilical endothelial (HUVEC) cells (newborn male, single donor) were obtained from Thermo Fisher Scientific (Waltham, USA). HUVECs and HEK293T cells were transfected and cultured as described in Method Details.

METHOD DETAILS

Materials

All materials were purchased from Sigma-Aldrich (Gillingham, UK) unless otherwise stated. FuGENE-HD, ONE-Glo™ luciferase and HaloTag® AF488 ligand were purchased from Promega Corporation (Wisconsin, USA). Opti-MEM was purchased from Thermo-Fisher Scientific (Massachusetts, USA). SNAP-Tag® AlexaFluor 647 (AF647) and AlexaFluor 488 (AF488) were purchased from New England BioLabs (Massachusetts, USA). $VEGF_{165}a$ was purchased from R&D Systems (Abingdon, UK). $VEGF_{165}a$-TMR was produced as described by Kilpatrick et al. (2017). BODIPY-CGP12177-TMR (Baker et al., 2003) was purchased from Molecular Probes (Oregon, USA). CGP12177 and ICI118551 were purchased from Tocris Bioscience (Bristol, UK). The cDNA construct expressing β-arrestin2-Venus-YFP was provided by Dr KDG Pfleger and generated as described in Kocan et al. (2008).

Molecular Biology

NLuc- and HaloTag-VEGFR Constructs

NLuc-VEGFR2 and HaloTag-VEGFR2 constructs were generated as described in Kilpatrick et al., 2017). Briefly, VEGFR2 was subcloned from a plasmid obtained from Origene (GenBank: NM_002253; Maryland, USA). VEGFR2 was cloned into a pF-sNnK CMV/neo vector (N1321; Promega Corporation, USA) encoding a fusion of the signal peptide sequence of IL-6 onto the N terminus of NanoLuc (NLuc). This resulted in open reading frames (ORFs) which encoded NLuc fused via a Gly-Ser-Ser-Gly(AIA) linker to the N terminus of VEGFR2 (termed NLuc-VEGFR2). For the N terminal HaloTag construct, VEGFR2 cDNA was cloned into a pFN21A CMV/neo flexi vector encoding a fusion of the signal peptide sequence of IL-6 onto the N terminus of HaloTag. The resultant ORFs encoded HaloTag fused to the N terminus of VEGFR2 via a EPTTEDLYFQSDN(AIA) linker (HaloTag VEGFR2).

NLuc- and SNAP-Tag- Adenosine A_3-Receptor Constructs

The generation of NLuc-A_3-receptor (Stoddart et al., 2015) and SNAP-A_3-receptor (Stoddart et al., 2014) pcDNA3.1 plasmids has been described previously. We generated NLuc-labeled adenosine receptor constructs by amplifying the full length sequence of NLuc luciferase (as provided by Promega Corporation in the pNL1.1 vector) and fusing it in frame with the membrane signal sequence of the $5HT_{3A}$ receptor within pcDNA3.1(+) to yield sig-NLuc. We then fused the full-length human sequence of the adenosine A3-receptor (obtained from Missouri S&T cDNA Resource Centre; www.cdna.org; GenBank: AY136749) with the methionine start signal removed, to the 3′ end of the sig-NLuc in pcDNA3.1(+). This gave the construct designated as NLuc-A_3 receptor. To generate N-terminal SNAP-tagged adenosine A_3 receptor, the methionine start signal was removed from cDNA encoding the full length A_3-receptor and subcloned into a pcDNA3.1-zeo (+) vector containing the $5HT_3$-receptor-derived signal sequence followed by the SNAP Tag sequence (New England Biolabs, Ipswich, MA, USA).

β_2-Adrenoceptor Constructs

The β_2-adrenoceptor cDNA sequence (obtained from Missouri S&T cDNA Resource Centre; www.cdna.org; GenBank: NM_000024.3) was PCR amplified to generate a β_2-adrenoceptor sequence that was in frame with the BamHI restriction site of sig-NLuc (Stoddart et al., 2015) and sig-SNAP (Gherbi et al., 2015), and changed the start codon (Met) of the β_2-adrenoceptor sequence to Leu. The primers used were forward 5'-CCGCCGGATCCCTGGGGCAACCCGGGAACG-3' and reverse 5'-GGCGGGAATTCTTACAGCAGTGAGTCATTTG-3'. The PCR product was then ligated in frame into pcDNA3.1(+) containing sig.SNAP (Gherbi et al., 2015) or sig-NLuc (Stoddart et al., 2015) using BamHI and EcoRI restriction enzymes. This created the plasmids sig-SNAP-ADRB2-pcDNA3.1(+) and sig-NLuc-ADRB2-pcDNA3.1(+). β_2-NLuc adrenoceptor in the pF-sNnK vector was obtained from Promega Corporation.

Nb80-GFP

The synthesis of Nb80 encoding cDNA was based on the amino acid sequence information published previously (Rasmussen et al., 2011). This sequence was first reverse translated into nucleotide sequence and then codon optimised for expression in human cells. The designed fragment was assembled via the Gibson reaction (Gibson et al., 2009) according to manufacture protocol (Gibson Assembly master mix, E2611S NEB) with the following set of overlapping primers:

Nb80Fwd1:5'ATGGGACAGGTGCAGCTGCAGGAGAGCGGCGGCGGCCTGGTGCAGGCCGGCGGCAGCCTGAGACTGAGCTGCGCCGCCAGCGGCAGCATCTTCAGCATCAACACCATGGG-3'; Nb80Rev2:5'TGCCCTTCACGCTGTTGGCGTAGTTGGTGCTGCCGCCGCTGTGGATGGCGGCCACCAGCTCTCTCTGCTTGCCGGGGGCCTGTCTGTACCAGCCCATGGTGTTGATGCTG-3'; Nb80Fwd3:5'CGCCAACAGCGTGAAGGGCAGATTCACCATCAGCAGAGACAACGCCGCCAACACCGTGTACCTGCAGATGAACAGCCTGAAGCCCGAGGACACCGCCGTGTACTACTGCA-3'; Nb80Rev4:5'GTGGTGGTGGTGGTGGTGGCTGCTCACGGTCACCTGGGTGCCCTGGCCCCAGTAGTCGTACTCGTACAGCACGGCGCCGTAGTCCTTCACGTTGCAGTAGTACACGGCGG-3'.

The Gibson reaction product was further PCR amplified with Nb80Fwd5 5'-CTTCGAACTAGTGCCGCCACCATGGGACAGGTGCA GCTGCAG-3' & Nb80Rev6 5'- GGTGGCACTAGTGACCGGTATGTGGTGGTGGTGGTGGTGGCTGCTC primers, digested with SpeI restriction enzyme and cloned in to SpeI linearized pSIN-eGFP-BSD plasmid (Dixon et al., 2011) resulting in the pSIN-Nb80-GFP expression vector.

CRISPR/Cas9 Guide and Donor Vector Cloning

Guide RNA construction was performed as described previously in the detailed protocol (Ran et al., 2013). Briefly, two guide sequences, sgRNA1: CCTGCCAGACTGCGCGCCAT and sgRNA2: TTGCCCCATGGCGCGCAGTC targeting the N-terminal region of *ADRB2* were designed using the CRISPR Design Tool (Hsu et al., 2013) (http://crispr.mit.edu/) and were ligated as complementary oligonucleotides into the pSpCas9(BB)-2A-Puro (PX459) expression construct (from Feng Zhang, Addgene plasmid # 62988) linearized by the restriction enzyme BbsI.

Primers used for sgRNA1 construction were:
forward 5'-CACCGCCTGCCAGACTGCGCGCCAT-3' and
reverse 5'-AAACATGGCGCGCAGTCTGGCAGG-3'
and for sgRNA2 were:
forward 5'-CACCGTTGCCCCATGGCGCGCAGTC-3' and
reverse 5'- AACGACTGCGCGCCATGGGGCAA-3'.

To introduce DNA encoding NLuc into the *ADRB2* locus a donor repair template was designed using the UCSC genome browser (http://genome.ucsc.edu/, Human genome assembly (GRCh38/hg38) (Kent et al., 2002). Homology arms, left (hg38 chr5:148826832-148826057) and right (hg38 chr5: 148826836-148827611), surrounding but not including the *ADRB2* start codon were synthesized as double stranded DNA by GeneArt (Invitrogen). A short linker was included between the homology arms to allow ligation of sig-NLuc (Stoddart et al., 2015) into the template using the restriction enzymes KpnI and BamHI. A mutation introduced during synthesis to remove an internal KpnI restriction site was then corrected by site-directed mutagenesis. The primers used were forward 5'-CAG ATGCACTGGTACCGGGCCACC-3' and reverse 5'- GGTGGCCCGGTACCAGTGCATCTG-3'. The donor template therefore resulted in cells expressing gene-edited sig-Nluc-β_2-adrenoceptor with the start codon (Met) of the β_2-adrenoceptor deleted.

Heterozygous in-frame insertion of NLuc into the *ADRB2* locus was observed by PCR of purified genomic DNA and verified by Sanger sequencing of overlapping PCR amplicons.

Primer sets used for PCR and sequencing were:
Amplicon 1, forward 5'-*TTCGGAGTACCCAGATGGAG*-3' and
reverse 5'- *GTCTTGAGGGCTTTGTGCTC*-3'.
Amplicon 2, forward 5'-*TTCGGAGTACCCAGATGGAG*-3' and
reverse 5'-ACAGGCCAGTGAAGTGATGA-3'.
Amplicon 3, forward 5'-GACAAGCTGAGTGTGCAGGA-3' and
reverse 5'- *GTCTTGAGGGCTTTGTGCTC*-3'
Primers in *italics* anneal outside of the donor repair template.

Cell Culture

All HEK293 cell lines used here were HEK293T cells grown in Dulbecco's Modified Eagle's Medium (DMEM 6429) supplemented with 10% fetal calf serum at 37°C/5% CO_2. All stable and transient transfections were performed using FuGENE HD according to the manufacturer's instructions. The NLuc-β_2-adrenoceptor stable HEK293 cell line was provided by Promega Corporation (Wisconsin, USA). Cell passaging was performed when cells reached 80% confluency using PBS (Lonza, Switzerland) and trypsin (0.25% w/v in versene; Lonza, Switzerland).

CRISPR/Cas9 genome-engineering of HEK293 cells was performed as described previously (White et al., 2017). Briefly, HEK293 cells were seeded into 6 well plates and incubated for 24h at 37°C/5% CO_2. At 60% confluency, cells were transfected with px459 sgRNA/Cas9 expression constructs and the donor repair template. Cells were cultured for 24h then treated with puromycin (0.3ug/ml) for 3 days to select for transfected cells. Following selection, cells were cultured without puromycin for 1 day then seeded into clear flat bottom 96-well plates at 1 cell per well and allowed to expand for 2-3 weeks. Single colonies were screened for luminescence following the addition of furimazine (10μM) using a PHERAStar FS plate reader. Positive clones were expanded before cells were collected for genotyping and sequencing.

Human umblical vein endothelial cells (HUVECs; passage 2-8) were grown in Medium 200 (ThermoFisher, USA) supplemented with LVES 50x large vessel endothelial cell supplement (ThermoFisher, USA) at 37°C/5% CO_2. Cell passaging was performed when cells reached 70% confluency using PBS (Lonza, Switzerland) and trypsin (0.25% w/v in versene; Lonza, Switzerland).

NanoBRET Assays to Determine Fluorescent Ligand Saturation Binding

HEK293 cells stably expressing full length cDNA encoding an N-terminal NLuc-tagged β_2-adrenoceptor (Stoddart et al., 2015) or NLuc-VEGFR2 (Kilpatrick et al., 2017) were seeded into poly-D-lysine coated white flat bottom 96 well plates (655089; Greiner Bio-One, Stonehouse, UK), and incubated for 24h at 37°C/5%CO_2. On the day of the assay, cells were washed and incubated with 1x HEPES Buffered Salt Solution (HBSS; 10mM HEPES, 10mM glucose, 146mM NaCl, 5mM KCl, 1mM $MgSO_4$, 2mM sodium pyruvate, 1.3mM $CaCl_2$; pH 7.2), pre-heated at 37°C. Cells were incubated with increasing concentrations of the appropriate

fluorescent ligand for β_2-adrenoceptor or VEGFR2 (BODIPY-CGP12177-TMR or VEGF$_{165}$a-TMR respectively) in HBSS for 60min at 37°C. Non-specific binding was defined using unlabelled subtype selective ligands (10μM propranolol or 10nM VEGF$_{165a}$ respectively). All VEGF incubations were performed using HBSS supplemented with 0.1% BSA. Following ligand incubation, 10μM of the NLuc substrate furimazine was added in the dark and plates left for 5min at room temperature. Sequential emission measurements were taken using a PHERAStar FS plate reader using 460nm (80nm bandpass; donor NLuc emission) and 610nm (longpass filter; fluorescent ligand emission) filters. Raw BRET ratios were calculated by dividing the 610nm emission (acceptor) by the 460nm emission (donor).

NanoBRET Saturation Assays to Investigate Receptor-Receptor Interactions

For homodimer studies, HEK293 cells were seeded into poly-D-lysine coated white flat bottom 96 well plates and incubated for 24h at 37°C/5% CO_2. At 70% confluency, cells were transiently transfected with a fixed concentration of N-terminal NLuc-tagged donor receptor constructs (0.05μg/well VEGFR2; 0.05μg/well β_2-adrenoceptor or A_3-receptor respectively) and increasing concentrations of N-terminal-tagged acceptor constructs (0.025-0.2μg/well HaloTag- VEGFR2; or 0.025-0.2μg/well SNAP Tag-β_2-adrenoceptor or A_3-receptor). When investigating the effect of ligand stimulation on the formation of VEGFR2 homodimers, a 1:1 (0.05:0.05μg/well) ratio of donor (NLuc-VEGFR2) to acceptor (HaloTag-VEGFR2) was also prepared. All transfections were performed using FuGENE HD in Opti-MEM according to the manufacturer's instructions. Empty p3.1zeo vector was used when necessary to ensure total cDNA concentrations were kept consistent across all wells. Cells were left to grow for a further 24h at 37°C/5% CO_2.

On the day of the assay, 24h post transfection, cells were incubated for 30min at 37°C/5%CO_2 with 0.2μM HaloTag AF488 or 0.2μM SNAP-Tag AF488 membrane impermeable substrate, prepared in serum-free DMEM. After incubation, cells were washed 3 times with HBSS pre-heated to 37°C. VEGFR2 homodimers, were stimulated with vehicle or 1nM VEGF$_{165}$a in HBSS supplemented with 0.1% BSA for 60min at 37°C. All other BRET homodimer pairs were incubated in vehicle (HBSS) for 60min at 37°C. When investigating whether VEGFR2 dimer formation was ligand dependent, NLuc-VEGFR2:HaloTag-VEGFR2 homodimers (0.05μg/well HaloTag-VEGFR2 transiently transfected into NLuc-VEGFR2 stable cell line) were stimulated with a concentration response course of VEGF$_{165}$a (0.25-10nM) for 60min at 37°C. 10μM of the NLuc substrate furimazine was then added to each well and plates left for 5min in the dark at room temperature. Sequential emission measurements were taken using a PHERAStar FS plate reader using 460nm (80nm bandpass; donor NLuc emission) and 535nm (60nm bandpass; HaloTag or SNAP-Tag emission) filters. Raw BRET ratios were calculated by dividing the 535nm emission (acceptor) by the 460nm emission (donor).

For heterodimer studies, HEK293 cells were seeded into poly-D-lysine coated white flat bottom 96 well plates and incubated for 24h at 37°C/5%CO_2. At 70% confluency, cells were transiently transfected with a fixed concentration of donor N-terminal tagged NLuc-VEGFR2 (0.05μg/well) and increasing concentrations of N-terminal-tagged acceptor receptor constructs (0.002-0.04μg/well or 0.025-0.2μg/well for SNAP-Tag-β_2-adrenoceptor or A_3-receptor respectively). All transfections were performed using FuGENE HD in Opti-MEM according to the manufacturer's instructions. Empty p3.1zeo vector was used when necessary to ensure total cDNA concentrations were kept consistent across all wells. Cells were left to grow for a further 24h at 37°C/5%CO_2. On the day of the assay, 24h post transfection, cells were incubated for 30min at 37°C/5%CO_2 with 0.2μM HaloTag AF488 or 0.2μM SNAP-Tag AF488 membrane impermeable substrate, prepared in DMEM/10% FCS. After incubation, cells were washed 3 times with HBSS pre-heated to 37°C. VEGFR2/combinations were stimulated with vehicle or 3nM VEGF$_{165}$a in HBSS/0.1% BSA for 60min at 37°C. For all other BRET combinations, cells were incubated in vehicle (HBSS) for 60min at 37°C. Following this, 10μM of the NLuc substrate furimazine was added to each well and plates left for 5min in the dark at room temperature. Sequential luminescent and fluorescent emission measurements were recorded using a PHERAStar FS plate reader as previously described for homodimer studies.

For studies using CRISPR/Cas9 edited cells, HEK293 expressing gene-edited Nluc-β_2-adrenoceptors were seeded into poly-D-lysine coated white flat bottom 96 well plates at a density of 15,000-20,000 cells/well and incubated for 24h at 37°C/5%CO_2. Cells were then transiently transfected with Halotag-VEGFR2 (0.01μg/well) plus empty p3.1zeo vector (0.09μg/well). Cells were left to grow for a further 24h at 37°C/5%CO_2. On the day of the assay, 24h post transfection, cells were incubated for 30min at 37°C/5%CO_2 with 0.2μM HaloTag AF488 prepared in DMEM/10% FCS. After incubation, cells were washed 3 times with HBSS pre-heated to 37°C and further incubated in vehicle (HBSS) for 60min at 37°C. Following this, 10μM of the NLuc substrate furimazine was added to each well and plates left for 5min in the dark at room temperature. Sequential luminescent and fluorescent emission measurements were recorded using a PHERAStar FS plate reader as previously described for homodimer studies.

For studies using HUVECs (passage 2-8), cells were passaged, counted and resuspended at 350,000 cells in Ingenio electroporation solution (100μl; Ingenio electroporation kit; Mirus Bio, Wisconsin, USA). The appropriate cDNA was added to this cell suspension (NLuc-VEGFR2/pcDNA3.1 zeo cDNA (2μg cDNA respectively per 0.1ml cell suspension) or NLuc-VEGFR2/ SNAP Tag-β_2-adrenoceptor (2μg cDNA respectively per 0.1ml cell suspension). HUVECs were then electroporated at room temperature using an Amaxa Nucleofector I (programme V-001) and immediately resuspended in Medium 200, plated onto white flat bottomed Grenier 96 well plates (Greiner Bio-One, 655089) and left to grow for 24h at 37°C/5%CO_2. On the day of the assay, HUVECs were incubated for 30min at 37°C/5%CO_2 with 0.2μM SNAP-Tag AF488 prepared in DMEM/10% FCS. After incubation, cells were washed 3 times with HBSS pre-heated to 37°C, 10μM of the NLuc substrate furimazine was added to each well and plates left for 5min in the dark at room temperature. Sequential luminescent and fluorescent emission measurements were recorded using a PHERAStar FS plate reader as previously described for homodimer studies (3600/3600 gain values) using a focal height of 4.2mm.

Widefield Bioluminescence Microscopy

Bioluminescence imaging was performed using an Olympus LV200 Wide field inverted microscope, equipped with a 60x/1.42NA oil immersion objective lens. HEK293 cells at a density of 70,000 cells/well were seeded into a 35mm MatTek dish split in 4-wells, containing a high tolerance 1.5μm coverslip. 24h later, cells were co-transfected with NanoLuc-tagged human VEGFR2 and SNAP-tagged human β_2-adrenoceptor cDNAs (0.4μg each). Before imaging, media was removed, and cells were incubated with either serum-free DMEM or 0.5μM SNAP-surface AF647 substrate and incubated for 30min at 37°C/5%CO_2. After incubation, cells were washed three times and incubated with HBSS containing 400nM furimazine substrate (Promega) at 37°C for 15min. Images were acquired by capturing sequential channels: 1) TRITC channel using an external LED lamp excitation (acceptor excitation; using 561/14nm excitation and a 647 long-pass emission filters, 200ms exposure time) ; 2) DAPI channel (donor emission; using a 438/24nm emission filter, 5sec exposure time); 3) CY5 channel (BRET-excited acceptor; using a 647 long-pass emission filter, 30sec exposure time). All images were acquired with gain set to 200. Images were exported using Fiji ImageJ version 1.52e software.

NanoBRET Assays to Investigate the Effects of Isoprenaline or $VEGF_{165}$a on Heteromer Formation

HEK293 cells were transiently transfected with a 1:2 cDNA ratio (0.05:0.1μg/well) of donor (NLuc-VEGFR2) to acceptor (SNAP Tag-β_2-adrenoceptor) constructs using FuGENE HD in Opti-MEM at a reagent to cDNA ratio of 3:1. Cells were then left to grow for a further 24h at 37°C/5% CO_2. On the day of the assay cells were incubated with 0.2μM SNAP-Tag AF488 membrane impermeable substrate, prepared in DMEM (30min at 37°C). Cells were then washed 3 times with HBSS and stimulated with fixed concentrations of isoprenaline or $VEGF_{165}$a for 60min at 37°C. $VEGF_{165}$a stimulations were performed in HBSS/0.1% BSA for 60min at 37°C. At the end of the assay course, 10μM of furimazine was added per well and plates read using a PHERAStar FS as previously described for homodimer studies.

NanoBRET Assays to Investigate Potential for Cooperativity across Putative Receptor Dimer Interfaces

HEK293 cells were transiently transfected with a 1:2 cDNA ratio (0.05:0.1μg/well) of donor (NLuc-VEGFR2 or NLuc-β_2-adrenoceptor) to acceptor (SNAP-Tag-β_2-adrenoceptor or HaloTag-VEGFR2) constructs using FuGENE HD in Opti-MEM at a reagent to cDNA ratio of 3:1. Cells were then left to grow for a further 24h at 37°C/5%CO_2. On the day of the assay, cells were co-stimulated with a fixed concentration of fluorescent ligand ($VEGF_{165}$a-TMR (1 or 2nM) or BODIPY CGP12177-TMR (15nM)) in the presence or absence of increasing concentrations of unlabeled subtype selective ligands (ICI,118551 (0.01nM-10μM); CGP12177 (0.01nM-10μM) or isoprenaline (0.1nM-100μM)). Nonspecific binding of fluorescent ligands was defined using a high concentration of unlabeled ligands (10nM $VEGF_{165}$a or 10μM propranolol). All ligand co-incubations were performed in HBSS for 60min at 37°C. 10μM of the NLuc substrate furimazine was added to each well and plates left for 5min in the dark at room temperature. Sequential emission measurements were taken using a PHERAStar FS plate reader using 460nm (80nm bandpass; donor NLuc emission) and 610nm (longpass filter; fluorescent ligand emission) filters. Raw BRET ratios were calculated by dividing 610m emission (acceptor) by 460nm emission (donor).

VEGFR2 Phosphorylation Assay

HEK293T cells were seeded at 15,000 cells/well in black flat-bottomed 96-well plates (Greiner Bio-One, 655090) pre-coated with poly-D-lysine (0.01mg/ml in PBS). Following 24 h, cells were transfected with HaloTag-VEGFR2 in the presence or absence of Snap-Tag-β_2-adrenoceptor (0.05μg/well of each) using FuGENE HD in serum free DMEM at a reagent to cDNA ratio of 3:1. Cells were then grown for another 24 hours (37°C/5% CO_2). On the day of the assay, cells were labeled using membrane impermeant SNAP-Tag AF647 substrate (0.2μM; 30min at 37°C) prepared in DMEM (30min at 37°C). Cells were then washed 3 times with HBSS and stimulated for 15 or 60 min with 3nM $VEGF_{165}$a, 100nM isoprenaline or both ligands simultaneously. The receptor tyrosine kinase inhibitor cediranib (1μM; Sequoia Research Products, UK) was used a negative control. Cells were washed with 100μl/well PBS, fixed with 3% paraformaldehyde (PFA)/PBS for 20min at room temperature), washed (3x5min PBS), permeabilised with 0.025% Triton-X-100 in PBS, washed (3x5min PBS) and incubated with 3% BSA/1% glycine/PBS to reduce non-specific binding (30min, room temperature). After washing (3x5min PBS), cells were blocked with 10% chick serum in PBS (30min, room temperature) and incubated at 4°C overnight with rabbit monoclonal anti-VEGFR2 phosphoY1212 (Cell Signalling, 2477) diluted 1:200 in 10% chick serum/PBS. Cells were washed (3x5min PBS) and incubated in the dark at room temperature with secondary antibody chick anti-rabbit AlexaFluor488 (Thermo Fisher, A21441). Nuclei were stained with 2mg/ml H33342 (15min, room temperature), washed and stored at 4°C in PBS. Cells were imaged using an ImageXpress Micro widefield high content imaging system (Molecular Devices, USA) with a 20x objective at 4 sites per well using FITC, DAPI and CY5 filters (exposure 25ms, 1500ms and 1500ms respectively).

HUVEC Proliferation Assay

HUVECs (passage 4-8) were seeded at 5,000 cells/well in black flat-bottomed 96-well plates (Greiner Bio-One, 655090) in 10% LVES/Medium 200. Following 24 h of cell growth at 37°C/5% CO_2, plating medium was replaced with Medium 200 containing 0.1% serum for 24 h. Cells were then stimulated with $VEGF_{165}$a (R&D Systems) at 3nM (in 0.1% serum/medium), Isoprenaline (100nM or 10μM) or both ligands simultaneously in the presence or absence of the receptor tyrosine kinase inhibitor cediranib (1μM; Sequoia Research Products, UK). Cediranib (1μM) was also used alone as a negative control. Following 48 h stimulation at 37°C/5% CO_2, cells were washed with 100μl/well PBS, fixed with 3% PFA/PBS (20 min, room temperature) and nuclei stained with 2mg/ml H33342

(15 min, room temperature). Nuclei were imaged using an ImageXpress Micro widefield high content imaging system (Molecular Devices, USA) with a 4x objective using a DAPI filter (4 sites per well, 25ms exposure time).

NFAT Luciferase Reporter Gene Assay

HEK293T cells stably expressed HaloTag-VEGFR2 or NanoLuc-VEGFR2, as well as ReLuc2P, a Firefly luciferase reporter gene inserted downstream of the NFAT reporter to monitor NFAT-induced gene transcription (Promega Corporation, USA). Cells were grown to 70-80% confluency and seeded in DMEM containing 10% Fetal Calf Serum at 25,000 cells per well in white 96-well plates (Greiner Bio-One, 655089) pre-coated with poly-D-lysine (0.1mg/ml in PBS). Following 24 h of cell growth at 37°C/5% CO_2, plating medium was replaced with serum free DMEM for a further 24 h. Cells were stimulated in duplicate wells with increasing concentrations of $VEGF_{165}a$ (R&D Systems) or vehicle (serum free DMEM containing 0.1% BSA) for 5 h at 37°C/5% CO_2. Assay medium was then replaced with 50μl/well serum free DMEM and 50μl/well One-Glo Luciferase reagent (Promega Corporation, USA) and equilibrated for 5 min to enable the reagent to react with luciferase and allow background luminescence to subside. Luminescence was then measured by a TopCount platereader (Perkin Elmer, UK). Data were normalised to vehicle (0%) and response to 10nM $VEGF_{165}a$ (100%) in each experiment and expressed as mean ± SEM. (5 independent experiments). Potency (EC_{50}) values were derived as described previously (Kilpatrick et al., 2017).

β-arrestin Recruitment Assays

HEK293 cells were seeded into poly-D-lysine coated white flat bottom 96 well plates and incubated for 24 h at 37°C/5%CO_2. At 70% confluency, cells were transiently co-transfected with 0.04μg/well of C-terminal Venus YFP-tagged β-arrestin2 and N-terminal Halo Tag-tagged VEGFR2, together with 0.01μg/well of C-terminal NLuc-tagged β_2-adrenoceptor, using FuGENE HD at a DNA:FuGENE ratio of 3:1, in Opti-MEM media. On the next day, Opti-MEM media was replaced with HBSS/0.1% BSA, and 10μL furimazine at 10μM was added in each well. Furimazine substrate was incubated for 5min at 37°C. After incubation, sequential emission measurements were taken for 4min before ligand treatment (10μM isoprenaline, 10nM $VEGF_{165}a$), using a PHERAStar FS plate reader. Continuous readings were taken every 1min for a total time of 45min, after ligand treatment, using 460nm (80nm bandpass; donor NLuc emission) and 535nm (60nm bandpass; β-arrestin2-Venus-YFP emission) filters. Raw BRET ratios were calculated by dividing the 535nm emission (acceptor) by the 460nm emission (donor).

Nanobody-80 Recruitment Assays

HEK293 cells stably expressing a pSIN-Nb80-GFP construct were seeded into poly-D-lysine coated white flat bottom 96 well plates and incubated for 24h at 37°C/5%CO_2. At 70% confluency, cells were transiently transfected with 0.025μg/well of C-terminal NLuc-tagged β_2-adrenoceptor and 0.025μg/well HaloTag-VEGFR2 or empty vector (pcDNA3.1), using FuGENE HD at a DNA:FuGENE ratio of 3:1, in Opti-MEM media. On the day after transfection, Opti-MEM media was replaced with HBSS/0.1% BSA, and 10μL of 10μM furimazine substrate was added to each well, and incubated for 5min at 37°C. After incubation, sequential emission measurements were taken for 5min before ligand treatment (10μM isoprenaline, 10nM $VEGF_{165}a$), using a PHERAStar FS plate reader. Continuous readings were taken every 1min for a total time of 45min. PHERAStar FS settings used were the same described for the β-arrestin recruitment assay.

For the isoprenaline concentration-response assay, HEK293 cells stably expressing Nb80-GFP cells were transfected as described for the time-course assay. On the day after transfection, Opti-MEM media was replaced with HBSS/0.1%BSA and incubated with increasing concentrations of isoprenaline (0.1nM to 100μM, in the presence or absence of 10nM $VEGF_{165}a$; $VEGF_{165}a$ treatment was added to cells co-transfected with HaloTag-VEGFR2, and not to cells transfected with the empty vector). Plates were incubated after ligand treatment for 30min at 37°C/without CO_2. After incubation, 10μM furimazine substrate was added to each well, and incubated for 5min. Sequential emission measurements were then taken using a PHERAStar FS plate reader, with the same settings used for the β-arrestin recruitment assay.

Live Cell Confocal Imaging

HEK293 cells were seeded at 20,000 cells per well on poly-D-lysine coated Nunc Lab-Tek 8 well plates (ThermoFisher Scientific) two days prior to imaging and cultured for 24 h at 37°C/5% CO_2. Cells were transiently transfected with 0.25μg per well of HaloTag-VEGFR2, SNAP Tag-β_2-adrenoceptor cDNA and grown for 24 h at 37°C/5% CO_2. Additionally, double transient transfections were performed with HaloTag- and SNAP-Tag receptor cDNA combinations (0.25μg per well total cDNA). All transfections were performed using FuGENE HD in Opti-MEM according to the manufacturer's instructions at a reagent to cDNA ratio of 3:1 and then grown for an additional 24 h at 37°C/5% CO_2. On the third day, cells were incubated with 0.5μM HaloTag AF660 membrane impermeant ligand and/or 0.5μM SNAP Tag AF488 membrane impermeant ligand for 30min at 37°C/5% CO_2 in DMEM. Cells were then washed three times with HBSS followed by vehicle addition or stimulation with either 10nM $VEGF_{165}a$ or 10μM Isoprenaline respectively for 60min at 37°C. For VEGF incubations, HBSS was supplemented with 0.1% BSA. Cells were imaged live at 37°C using a Zeiss LSM710 fitted with a 63x Plan Apochromat oil objective (1.4NA) using Argon488 (AlexaFluor488; 496-574nm band pass; 3% power) and/or HeNe excitation (AlexaFluor647; 621-759nm bandpass; 20% power) using a 488/561/633 beamsplitter with a pinhole diameter of 1 Airy unit. All images were taken at 1024x1024 pixels per frame with 8 averages.

Rab5 Immunolabelling

HEK293 cells were seeded at 300,000 cells/well on poly-D-lysine coated 18x18mm 1.5H coverglasses (474030-9000-000; Zeiss, Germany) and cultured for 24 h at 37°C/5% CO_2. Double transient transfections were performed with HaloTag-VEGFR2 and SNAPTag β_2-adrenoceptor cDNA (3µg per well total cDNA). All transfections were performed using FuGENE HD in OptiMEM according to the manufacturer's instructions at a 3:1 reagent to cDNA ratio. Cells were grown for a further 24 h at 37°C/5% CO_2. Cells were then incubated with 0.2µM HaloTag AF488 membrane impermeant ligand and 0.2µM SNAPTag AF647 membrane impermeant ligand for 30min at 37°C/5% CO_2 in serum free DMEM/0.1% BSA. Coverslips were washed three times with HBSS/0.1% BSA and then stimulated with vehicle, 10nM $VEGF_{165}$a, 10µM isoprenaline or both $VEGF_{165}$a and 10µM isoprenaline simultaneously (30min at 37°C in HBSS/0.1% BSA). Cells were then washed with PBS, fixed using 3% paraformaldehyde/PBS (20min at room temperature) and permeabilised using Triton-X-100 (0.025% in PBS). Cells were washed extensively three times with PBS between fixation and permeabilisation. To minimize non specific binding, cells were incubated with 3% BSA/1% glycine in PBS (30min at room temperature) and then blocked using 10% donkey serum in PBS (30min at room temperature). Cells were then labeled with primary antibody diluted 1:100 in 10% donkey serum at 4°C overnight (rabbit monoclonal antibody anti Rab5 (35475); New England Biolabs, USA). On the following day, cells were washed three times with PBS and incubated with 1:500 dilution in 10% donkey serum/PBS of secondary antibody (donkey anti rabbit IgG AlexaFluor 568 (10617183); Thermo Fisher Scientific, USA) for 1h at room temperature in the dark. Coverslips were then washed three times in PBS and nuclei labeled using bisBenzimide H 33342 trihydrochloride (H33342; Sigma Aldrich, USA (B2261) for 20min at room temperature. Coverslips were mounted onto microscope slides using Prolong Glass (P36965; Thermo Fisher Scientific, USA) and imaged using a Zeiss LSM880 fitted with a PlanApochromat 63x/1.4 NA oil objective using simultaneous multitrack settings. HaloTag VEGFR2 was imaged using a Argon488 laser line (AlexaFluor488; 490-571nm bandpass; 3% power) using a 488nm beamsplitter (1.43 Airy unit pinhole diameter, 1µm slice), Rab5 immunolabelling imaged using a DPSS 561-10 laser line (AlexaFluor561; 570-642nm; 3% power) using a 458/561nm beamsplitter (1.21 Airy unit, 1µm slice) and SNAP Tag β_2AR imaged using HeNe633 laser line (AlexaFluor633; 638-747nm; 15% power) using a 488/561/633nm beamsplitter (1 Airy unit, 1µm slice). All images were taken at 1024x1024 pixels per frame (0.131µm per pixel) with 8 averages.

Structured Illumination Microscopy

HEK293 cells were grown at a density of 200,000 cells on poly-D-lysine coated 18x18mm 1.5H coverglasses (474030-9000-000; Zeiss, Germany) and cultured for 24hr at 37°C/5% CO_2. Double transient transfections were performed with HaloTag-VEGFR2 and SNAP-Tag β_2-adrenoceptor (3µg per well total cDNA). All transfections were performed using FuGENE HD in OptiMEM according to the manufacturer's instructions at a reagent to cDNA ratio of 3:1. Cells were then grown for an additional 24hr at 37°C/5% CO_2. On the third day, cells were incubated with 1µM HaloTag AF488 membrane impermeant ligand and/or 1µM SNAP-Surface AF647 membrane impermeant ligand for 30min at 37°C/5% CO_2 in DMEM/10% FCS. Cells were then washed three times with HBSS and then stimulated with either vehicle, 10nM $VEGF_{165}$a or 10µM Isoprenaline respectively for 30min at 37°C. For VEGF incubations, HBSS was supplemented with 0.1% BSA. All cells were then fixed using 3% PFA/PBS (10min at room temperature), washed with PBS and mounted onto slides using a 1:1 mixture of CFM3 (E17979-20; CitiFluor, USA) and Prolong Glass anti-fade reagent (P36965; Thermo Fisher Scientific, USA). Slides were imaged using a Zeiss ELYRA PS.1 microscope using a Plan Apochromat 63x/1.4 oil DIC M27 objective with Zeiss Immersol™ 518F (30°C) oil (Zeiss, Germany). Multitrack imaging with HaloTag-VEGFR2 imaged using bandpass 495-550 plus longpass 750 filter at 5% laser power with 150ms exposure time (28µm grating) and SNAP-Tag β_2-adrenoceptor with a long-pass 655 filter at 8% laser power with 150ms exposure time (42µm grating). All images were acquired at 1024x1024 frame size over 5 rotations as a Z stack of 30-40 slices. Images were manually processed with consistent raw scaling between and within experiments (sectioning of 100x83x93; Zen Black 2012, Zeiss, Germany). TetraSpeck™ microspheres (0.1 µm; T7279, Thermo Fisher Scientific, USA) were included in each experiment to allow X/Y/Z channel alignment correction in image processing. TetraSpeckTM microspheres are stained throughout with four different fluorescent dyes, yielding excitation/emission peaks of 365/430 nm (blue), 505/515 nm (green), 560/580 nm (orange), and 660/680 nm (red).

QUANTIFICATION AND STATISTICAL ANALYSIS

Analysis of ligand-binding curves was undertaken using Prism 7 software (GraphPad, San Diego, USA). Data analysis performed for NanoBRET receptor-ligand saturation binding assays is described in detail in (Stoddart et al., 2015). Briefly, total and non-specific saturation binding curves were simultaneously fitted using the following equation:

$$SB = \frac{(B_{max} \times [L])}{([L] + K_D)} + ((M \times [L]) + C),$$

where L is the concentration of fluorescent ligand, B_{max} is the maximal specific binding, K_D is the dissociation constant of the fluorescent ligand, M is the slope of the non-specific binding element and C is the intercept with the y axis.

Data analysis for receptor-receptor NanoBRET saturation assay was performed using emission measurements through two wavelength windows. Baseline-corrected BRET ratios were calculated as shown below:

$$Baseline\ corrected\ BRET\ ratio = [(emission\ at\ 535nm)/(emission\ at\ 460nm) - Cf],$$

where *Cf* corresponds to *(emission at 535nm/emission at 460nm)* for the NLuc-construct expressed alone in the same experiment.

Agonist concentration-response data were fitted using Prism 7 software (GraphPad, San Diego, USA) to the following equation:

$$Response = \frac{E_{MAX} + [A]}{EC_{50} + [A]}$$

Where E_{MAX} is the maximum response, EC_{50} is the concentration of agonist required to produce 50% of the maximal response and (A) is the agonist concentration.

Dissociation constants (K_D) of antagonists were also calculated from the shift of the agonist concentration response curves in the presence of a fixed concentration of antagonist using the following equation:

$$CR = 1 + [B]/K_D$$

where CR (concentration ratio) is the ratio of the agonist concentration required to stimulate an identical response in the presence and absence of the fixed concentration of antagonist (B).

Statistical analysis was largely performed on repeated experiments with matched experimental conditions using two-way ANOVA with Dunnett's multiple comparison tests. Mean data from 5-8 independent experiments were normally analysed. Specific information on the number of separate experiments performed, and the number of replicates obtained in each individual experiment, is provided in each figure legend. Where time course experiments were analysed, two-way ANOVA was used with repeated measures and Bonferroni's multiple comparison tests. Otherwise, one way Analysis of Variance (ANOVA) significance tests were performed using baseline corrected BRET ratios with Tukey's multiple comparison tests. Experiments using CRISPR/Cas9 genome-edited cells used unpaired t-test for individual experiments and paired two-tailed t-test for the mean data obtained in each experiment. In all cases, differences were considered significant at $p < 0.05$ and the data were assumed to be normally distributed.

The extent of HaloTag-VEGFR2 and SnapTag β_2-adrenoceptor co-localisation in SIM images was analysed using the Fiji (ImageJ; version 2.0.0-rc-69/1.52i) CoLoc2 analysis programme. Circular regions of interest (ROIs; 6 ROI for spectral bead images and 12-15 OIS for all experimental conditions) were placed on either the plasma membrane or intracellular regions of acquired images. Pearson's correlation cofficients were obtained and averaged across all ROIs, with values then pooled and expressed as mean ± SEM.

DATA AND SOFTWARE AVAILABILITY

GraphPad Prism 7.02 (San Diego, CA, USA) was used to analyse the quantified data and produce the graphs. MetaXpress 2.0 (Molecular Devices, USA) was used to quantify HUVEC proliferation and VEGFR2 phosphorylation following high content imaging on the IX Micro widefield platereader. SIM images were analysed for extent of fluorophore colocalisation using the Fuji (ImageJ; version 2.0.0-rc-69/1.52i) CoLoc2 analysis programme.

Cell Chemical Biology, Volume *26*

Supplemental Information

Complex Formation between VEGFR2 and the β_2-Adrenoceptor

Laura E. Kilpatrick, Diana C. Alcobia, Carl W. White, Chloe J. Peach, Jackie R. Glenn, Kris Zimmerman, Alexander Kondrashov, Kevin D.G. Pfleger, Rachel Friedman Ohana, Matthew B. Robers, Keith V. Wood, Erica K. Sloan, Jeanette Woolard, and Stephen J. Hill

Supplementary Information.

Complex formation between VEGFR2 and the β_2-adrenoceptor.

Laura E. Kilpatrick[1,2♯], Diana C. Alcobia[1,2,7♯], Carl W. White[1,2,5], Chloe J Peach[1,2], Jackie R Glenn[1,2], Kris Zimmerman[3]; Alexander Kondrashov[4], Kevin D. G. Pfleger[5,6], Rachel Friedman-Ohana[3], Matthew B. Robers[3], Keith V. Wood[3], Erica K Sloan[7,8,9], Jeanette Woolard[1,2,*], Stephen J. Hill[1,2,*]

Supplementary Table 1 (Related to Figure 2).

Maximum BRET values ($BRET_{max}$; baseline corrected) for GPCR-GPCR and VEGFR2-GPCR heterodimers.

**Homodimers**				
**Donor**	**Acceptor**	**$BRET_{MAX}$**		**n**
NLuc-A_3R	SNAP-A_3R	0.021 ± 0.001	$p < 0.001$	5
NLuc-β_2AR	SNAP-β_2AR	0.088 ± 0.012	$p < 0.001$	5
**Heteromers**				
**Donor**	**Acceptor**	**$BRET_{MAX}$**		**n**
NLuc-VEGFR2	SNAP-A_3R	-	NS	5
NLuc-VEGFR2	SNAP-β_2AR	0.015 ± 0.002	$p< 0.01$	5

$BRET_{MAX}$ values for homodimers and NLuc-VEGFR2-heterodimers are taken from individual experiments summarized in Figure 2. Values are mean ± SEM from n separate experiments. Statistical analysis was performed using One-way ANOVA with Tukey's test and compares the $BRET_{MAX}$ value to the equivalent baseline BRET signal. NS=not significant.

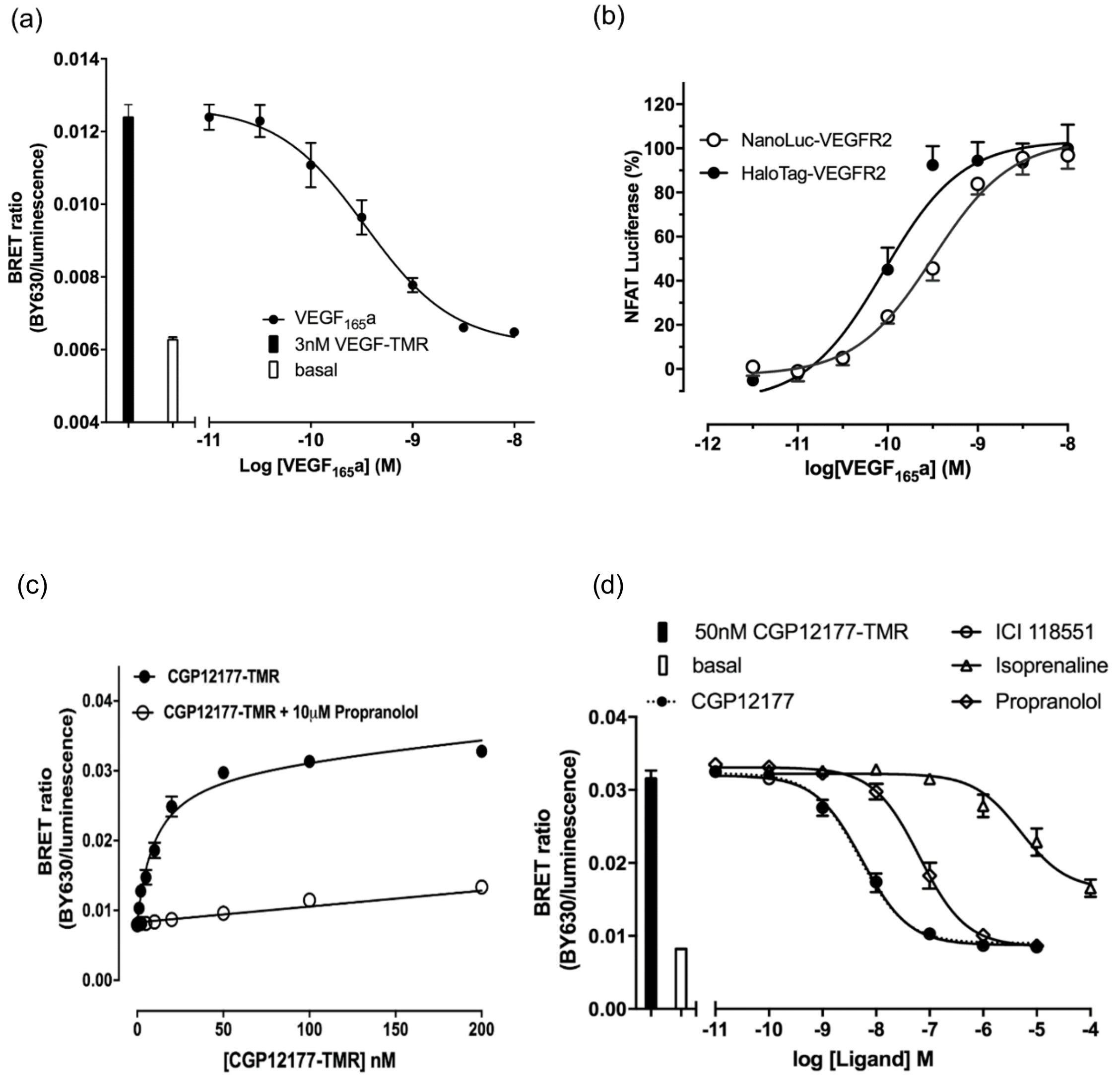

Supplementary Figure S1. (Related to Figures 1 and 2). (a,b) Characterization of the interaction between VEGF$_{165}$a and VEGFR2 using ligand binding studies and functional responses in HEK293 cells. (a) Inhibition of the binding of 3nM VEGF$_{165}$a-TMR to NLuc-VEGFR2 by increasing concentrations of VEGF$_{165}$a. Data represent mean ± SEM from seven separate experiments, each performed in duplicate. (b) NFAT luciferase production in HEK293T cells stably expressing HaloTag-VEGFR2 or NLuc-VEGFR2 in response to stimulation (5h) by VEGF$_{165}$a. Data are mean ± SEM (5 independent experiments, duplicate wells) expressed as a percentage of the response to 10nM VEGF$_{165}$a. (c, d) Characterization of the ligand binding properties of BODIPY CGP12177-TMR. (c) NanoBRET saturation binding curves obtained for BODIPY CGP12177-TMR binding to NLuc-β$_2$-adenoceptors. Non-specific binding (open circles) was established by the addition of 10μM Propranolol. Values are mean ± SEM from five separate experiments each performed in triplicate. pK_D of CGP12177-TMR was 7.98 ± 0.11 (n=5). (d) Inhibition of the binding of 50nM BODIPY CGP12177-TMR to NLuc-β$_2$-adrenoceptor by increasing concentrations of β-adrenoceptor

ligands. Data represent mean ± SEM from six separate experiments, each performed in duplicate. pK_i values obtained for ICI 118551, propranolol, CGP12177 and isoprenaline were 9.06 ± 0.12, 7.97 ± 0.12, 8.88 ± 0.21 and 6.63 ± 0.24 respectively (n=6).

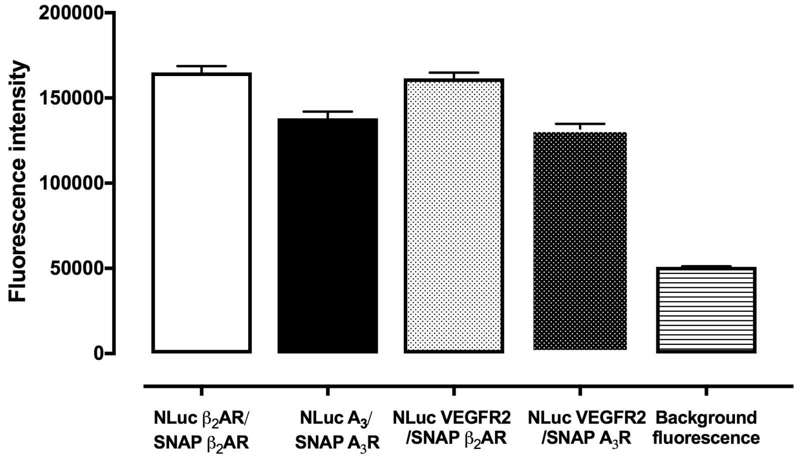

Supplementary Figure S2 (related to Figure 2). Comparison of expression levels of SNAP-tagged-β2-adrenoceptors and SNAP-tagged adenosine A3-receptors in transient transfection experiments involving NLuc-tagged GPCRs and NLuc-VEGFR2. Transient expression was performed with 50ng/well NLuc VEGFR2 or NLuc-GPCR and 100ng/well of SNAP-tag β2-adrenoceptor or adenosine A3-receptor cDNA. Background fluorescence was determined in cell transfects with 50ng/well NLuc-VEGFR2 and 100ng/well of pcDNA3.1-Zeo vector. Fluorescent intensity was determined on the PheraStar plate reader using excitation (485nm)/emission (520nm) filters from a 3x3 well scan matrix. Values represent mean ± SEM of 16 replicates in each of three independent experiments.

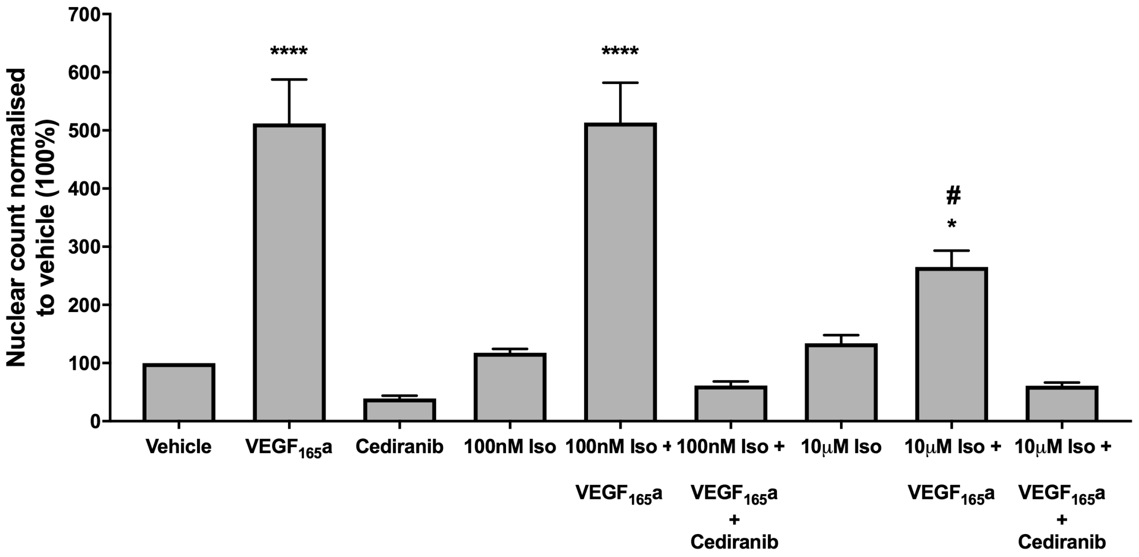

Supplementary Figure S3. (Related to Figure 4c). Effect of $VEGF_{165}a$ and isoprenaline (Iso) stimulation on proliferation of human umbilical vein endothelial cells (HUVECs). HUVECs were serum starved in Medium 200/0.1% LVES serum for 24h prior to stimulation. On the day of the assay, HUVECs were stimulated in triplicate with $VEGF_{165}a$ (3nM), isoprenaline (100nM or 10µM) or both ligands simultaneously in the presence or absence of the receptor tyrosine kinase inhibitor cediranib (1µM) for 48hr at 37°C/5% CO_2). Cediranib alone was used a negative control. HUVECs were then fixed using 3% paraformaldehyde in PBS and nuclei stained using H33342. Cells were imaged using a widefield IX Micro platereader and nuclei counted. Data are expressed as mean ± SEM pooled from 4 independent experiments and normalized to vehicle responses (100%). **** p< 0.0001 or * p<0.05 compared to vehicle and # p<0.0001 VEGF165a v Iso+VEGF165a (one-way ANOVA with Tukey's multiple comparison test).

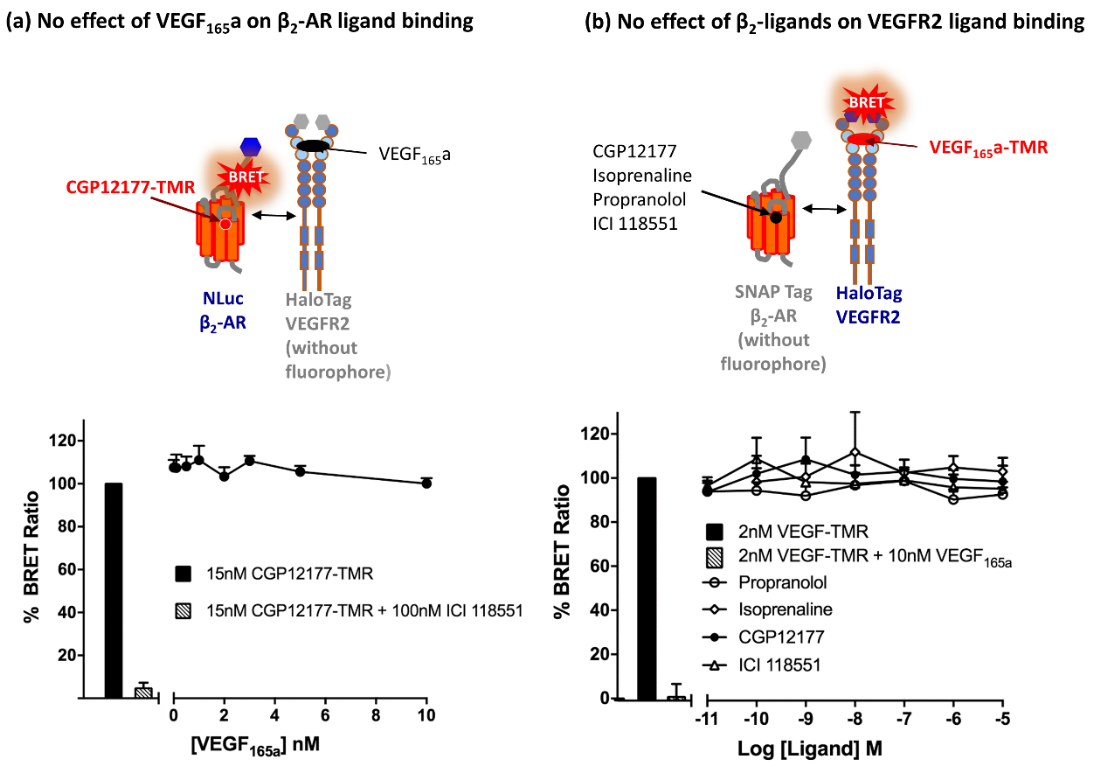

Supplementary Figure S4. **(Related to Figure 2).** No evidence for cooperativity across β_2-adrenoceptor-VEGFR2 interfaces provided by ligand-binding studies using a fluorescent ligand for the β_2-adrenoceptor (a; BODIPY CGP 12177-TMR) or the fluorescent $VEGF_{165}$a-TMR ligand for VEGFR2 (b; $VEGF_{165}$a-TMR). HEK293 cells were transiently transfected with a 1:2 cDNA ratio (0.05:0.1µg/well) of donor (NLuc-VEGFR2, NLuc-β_2-adrenoceptor) to acceptor (SNAP-Tag-β_2-adrenoceptor or HaloTag-VEGFR2) constructs. (a) NLuc-β_2-adrenoceptor and HaloTag-VEGFR2; (b) NLuc-VEGFR2 and SNAP-Tag-β_2-adrenoceptor. BRET was measured between donor NLuc and acceptor fluorescent ligands. Data are mean ± SEM from five separate experiments, each performed in duplicate.

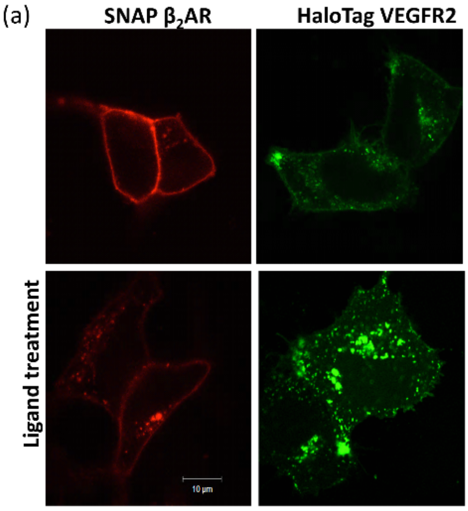

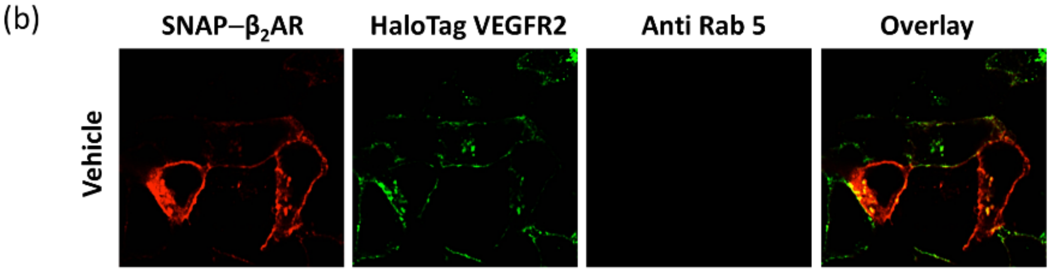

Supplementary Figure S5. (Related to Figure 6). (a) Live cell imaging showing the effect of agonist stimulation on the cellular location of β_2-adrenoceptors or VEGFR2 when expressed alone. Confocal imaging (Zeiss LSM 710) of live HEK293 cells transiently transfected with 0.25µg/well of SNAP-Tag-β_2AR or HaloTag VEGFR2 cDNAs, under unstimulated conditions (vehicle) or after treatment with 10µM isoprenaline (SNAP-β_2AR) or 10nM VEGF$_{165}$a ligands (HaloTag VEGFR2; 60min at 37°C). (b) Control experiments of secondary antibody only labelling of Rab 5 early endosomes. HEK293 cells transiently co-transfected with 0.5µg/well HaloTag VEGFR2 (green) and 0.5µg/well SNAP-Tag β_2AR (red) cDNAs, under unstimulated conditions (vehicle) (30min at 37°C). Cells were fixed using 3% PFA/PBS, permeabilised using Triton-X-100 (0.025% in PBS). Cells were then incubated with a secondary antibody (donkey anti rabbit AlexaFluor 546) in the absence of the primary antibody specific for Rab 5 endosomal compartments (rabbit anti Rab 5 monoclonal antibody). The overlay of HaloTag VEGFR2 and SNAP-Tag β_2AR expression with anti-Rab 5 labelling is shown. Data are representative of 3 individual experiments and were performed in parallel with those in Figure 6b.

(a)

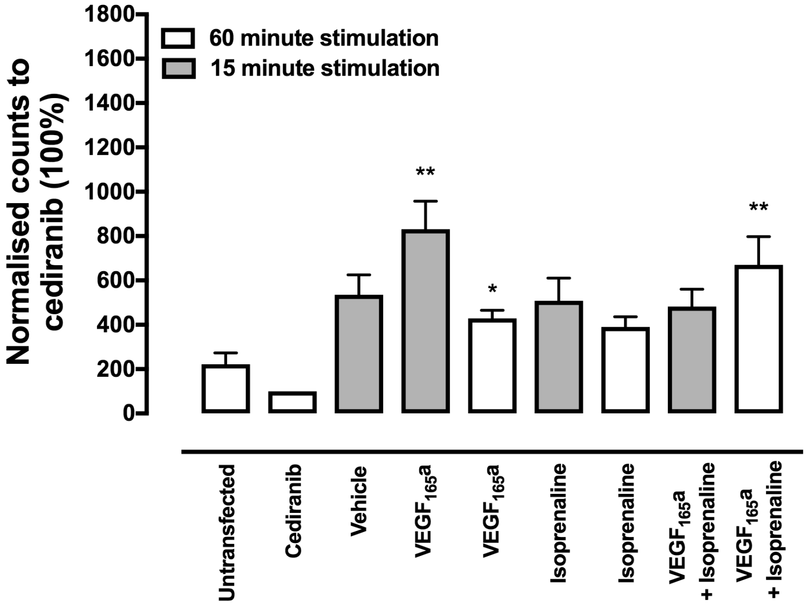

(b)

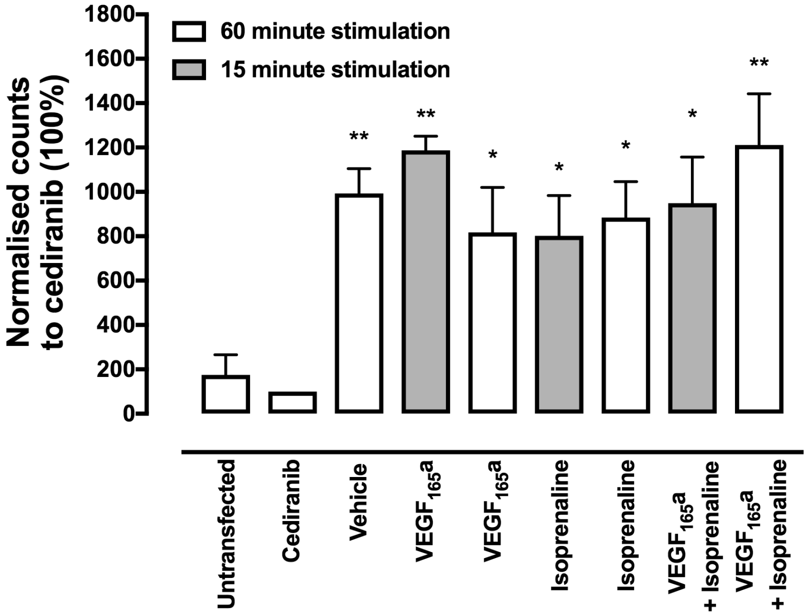

Supplementary Figure S6 (related to Figure 7). Probing the effect of isoprenaline stimulation on VEGFR2 activation status. HEK293 cells were transiently transfected with (a) HaloTag VEGFR2 or (b) HaloTag VEGFR2 and SNAP-Tag β_2-adrenoceptors (0.05µg/well of each construct) and stimulated with $VEGF_{165}a$ (3nM), isoprenaline (100nM) or both ligands simultaneously for 15 (grey bars) or 60min (white bars) at 37°C (5 replicates per stimulation). The receptor tyrosine kinase inhibitor cediranib was used as a negative control. Cells were fixed using 3% PFA/PBS and permeabilised using Triton-X-100 (0.025%). Immunolabelling

of the VEGFR2 tyrosine residue Y1212 was used to confirm the activation status of VEGFR2. Cell nuclei were stained using H33342 and images acquired using the IX Micro widefield platereader. Data were normalized as fold increase over cediranib responses (100%; mean ± SEM) and are pooled from 3 independent experiments. * ($p<0.05$) or ** ($p<0.01$) Compared to the cediranib control (One-way ANOVA with post hoc Tukey test).
